# Computation‐Guided Dual‐Site Electrocatalysts for Record‐Performance Nitrite‐to‐Ammonia Conversion

**DOI:** 10.1002/advs.202520683

**Published:** 2025-12-17

**Authors:** Hui Zhang, Haiyan Duan, Donglin Han, Zhenlin Wang, Xingchi Li, Dengchao Peng, Lupeng Han, Tianting Pang, Evangelina Pensa, Wenqiang Qu, Yongjie Shen, Haotian Wang, Wei Ren, Ming Xie, Emiliano Cortés, Dengsong Zhang

**Affiliations:** ^1^ Innovation Institute of Carbon Neutrality International Joint Laboratory of Catalytic Chemistry State Key Laboratory of Advanced Special Steel Department of Chemistry College of Sciences Shanghai University Shanghai 200444 P. R. China; ^2^ Nanoinstitute Munich Faculty of Physics Ludwig‐Maximilians‐Universität (LMU) 80539 Munich Germany; ^3^ Materials Genome Institute Qianweichang College Shanghai University Shanghai 200444 P. R. China; ^4^ Department of Chemistry University of Toronto 80 St. George Street Toronto ON M5S 3H6 Canada; ^5^ Institute for Chemical Reaction Design and Discovery (WPI‐ICReDD) Hokkaido University Sapporo 001–0021 Japan; ^6^ Department of Chemical Engineering University of Bath Bath BA2 7AY UK

**Keywords:** ammonia synthesis, dual‐site catalysts, electrocatalysis, first‐principles calculation, nitrite reduction

## Abstract

Designing catalysts that can simultaneously accelerate reactant activation and hydrogenation remains a central challenge in electrochemical ammonia synthesis. Here, a computation‐guided, dual‐site electrocatalyst design strategy that bridges first‐principles theory with device‐level validation is reported. Guided by density functional theory, Cu‐doped ZnO is identified as an optimal dual‐site platform: Cu sites upshift the Zn d‐band center, strengthening ^*^NO_2_ adsorption and enabling facile deoxygenation, while ZnO sites promote water dissociation to supply protons at the reaction interface. This cooperative synergy precisely tunes nitrite activation and hydrogenation kinetics, suppressing competing hydrogen evolution. The resulting catalyst achieves a record NH_3_ yield of 552.16 mg h^−1^ cm^−2^ with 87.9% Faradaic efficiency in a membrane electrode assembly—4× and 18× higher than flow‐ and H‐cell configurations, respectively. Operando spectroscopy confirms the predicted mechanism, demonstrating a theory‐to‐device workflow that replaces trial‐and‐error with predictive catalyst design. This approach establishes a generalizable paradigm for developing advanced electrocatalysts for sustainable chemical transformations.

## Graphical abstract

Designing Cu doped ZnO catalysts for NO_2_RR via first principles calculation and boosting electrocatalytic nitrite reduction through Cu‐ZnO synergistic deoxygenation, hydrogenation, and NH_3_ desorption. Cu doping elevates the d‐band center of Zn, strengthening ^*^NO_2_ binding and deoxygenation, while ZnO sites regulate water adsorption and dissociation, providing H, establishing a proton‐rich microenvironment, and accelerating interfacial mass transfer kinetics.

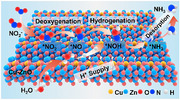

## 1 Introduction

Ammonia (NH_3_) is a crucial feedstock not only for the chemical industry but also a promising next‐generation hydrogen storage medium and carbon‐neutral energy carrier.^[^ ^1^, ^2^, ^3^ ^]^ Currently, the industrial Haber–Bosch process for ammonia production requires high temperatures and high pressures, resulting in excessive energy consumption and carbon emissions.^[^ ^4^ ^]^ Electrocatalytic nitrogen reduction reaction (NRR) is a promising route for sustainable ammonia synthesis, but still hindered by the strong N≡N triple bond (941 kJ·mol^−1^) and competitive hydrogen evolution reaction (HER).^[^ ^5^, ^6^, ^7^, ^8^ ^]^ Recently, electrocatalytic nitrite reduction reaction (NO_2_RR) has emerged as a novel alternative due to a much lower dissociation energy for N═O bond (only 204 kJ mol^−1^),^[^ ^9^, ^10^ ^]^ higher solubility coupled with fast reaction kinetics at the liquid‐solid interface. Moreover, nitrite (NO_2_^−^) serves as a key intermediate in the nitrogen cycle conversion of nitrate, thus offering fundamental insights into complete nitrate reduction mechanisms. It is also a common pollutant in severely contaminated water resources, posing substantial risks to environmental and human health.^[^ ^11^, ^12^ ^]^ Therefore, the electrocatalytic conversion of NO_2_^−^ into value‐added NH_3_ offers substantial environmental and economic benefits.^[^ ^13^, ^14^, ^15^ ^]^ The NO_2_RR process is a complex process demanding six electrons and seven protons (NO_2_^−^ + 6e^−^ + 7H⁺ → NH_3_ + 2H_2_O), and involving chemical activation of NO_2_^−^ and subsequent hydrogenation steps.^[^ ^16^, ^17^ ^]^ Therefore, engineering efficient catalysts to promote electron transfer and proton supply is crucial for achieving high NH_3_ yield and Faradaic Efficiency (FE).

Transition metal‐based catalysts are conventionally used for NO_2_RR owing to their simple preparation, low cost, reusability, and durability.^[^ ^18^, ^19^, ^20^, ^21^, ^22^ ^]^ Currently, various strategies, including morphological size control,^[^ ^23^ ^]^ lattice defect construction,^[^ ^24^ ^]^ second metal introduction,^[^ ^25^ ^]^ and oxygen vacancy formation,^[^ ^26^ ^]^ have been explored to further improve their NO_2_RR performance. For example, Xiao and coworkers reported Cu_6_Sn_5_ alloy as an electrocatalyst that efficiently synthesized ammonia from nitric oxide, and the kinetic barriers of protonation were invariably low over a range of Cu_6_Sn_5_‐derived surface structures.^[^ ^27^ ^]^ Jiang et al. revealed that the electrosynthesis of ammonia is dramatically accelerated by introducing oxygen‐vacancy‐rich bismuth nanocrystals, which optimize the adsorption energetics of key ^*^NO intermediates.^[^ ^28^ ^]^ However, the conventional method in experiments to screen effective NO_2_RR catalysts is still a “trial‐and‐error” strategy on a case‐by‐case basis. To assist the experimental development and smart catalyst design, first‐principles calculations based on mechanistic investigations play a complementary and even decisive role. For instance, Cheng et al. combine the first‐principles calculation with a microkinetic model, screening 172 bimetallic alloys considering the hydrogen covering effect on the surface in the selective hydrogenation reaction.^[^ ^29^ ^]^ The rapid growth of computational screening can quickly evaluate the crucial factors and predict the potential candidates for NO_2_RR applications. It is envisioned that the computationally guided design of metal‐based catalysts would be a rational, cost and time‐saving approach for screening out the desired catalysts with superior NO_2_RR performance. However, it is rarely demonstrated in NO_2_RR.

Herein, based on first‐principles calculations that considered ^*^H supply, ^*^NO hydrogenation, and ^*^NH_3_ desorption, Cu‐ZnO dual site catalysts were systematically identified to be good candidates for NO_2_RR. The Cu‐ZnO catalysts can ultimately enhance catalytic selectivity and reduce the variety of products via easier ^*^NO hydrogenation and ^*^NH_3_ desorption. Then, we synthesized Cu‐ZnO catalysts and explored their NO_2_RR performance using an H‐cell, a flow cell, and a membrane electrode assembly (MEA) reactor, respectively. The theoretically established screen was precisely validated through experiments. The Cu‐ZnO catalysts achieved an ammonia yield of 552.16 mg h^−1^ cm^−2^ with a FE of 87.9% in 0.5 m nitrite using the MEA reactor. This represents a 4‐fold improvement over flow cell (133.45 mg h^−1^ cm^−2^ with a FE of 89.1%) and an 18‐fold enhancement compared to H‐cell configurations (29.75 mg h^−1^ cm^−2^ with a FE of 92%), significantly surpassing most reported NO_2_RR electrocatalysts. The Cu‐ZnO catalysts also enable a long‐time electrolysis test of over 100 h with steady current. Mechanistic studies combining operando spectroscopy and DFT reveal that Cu doping upshifts the d‐band center of Zn, enhancing ^*^NO_2_ binding and facilitating deoxygenation, while ZnO sites modulate water dissociation to establish a proton‐rich interfacial microenvironment. This Cu doped ZnO dual‐site catalysis effectively tailors the nitrite activation and hydrogenation kinetics, thereby suppressing the hydrogen evolution reaction (HER) and maximizing NH_3_ yield. This study pioneers a computation‐to‐device workflow for NO_2_RR, replacing empirical “trial‐and‐error” with mechanism‐guided catalyst design, which will provide a rational design paradigm for developing advanced NO_2_RR electrocatalysts.

## 2 Results and Discussion

**Figure 1:**
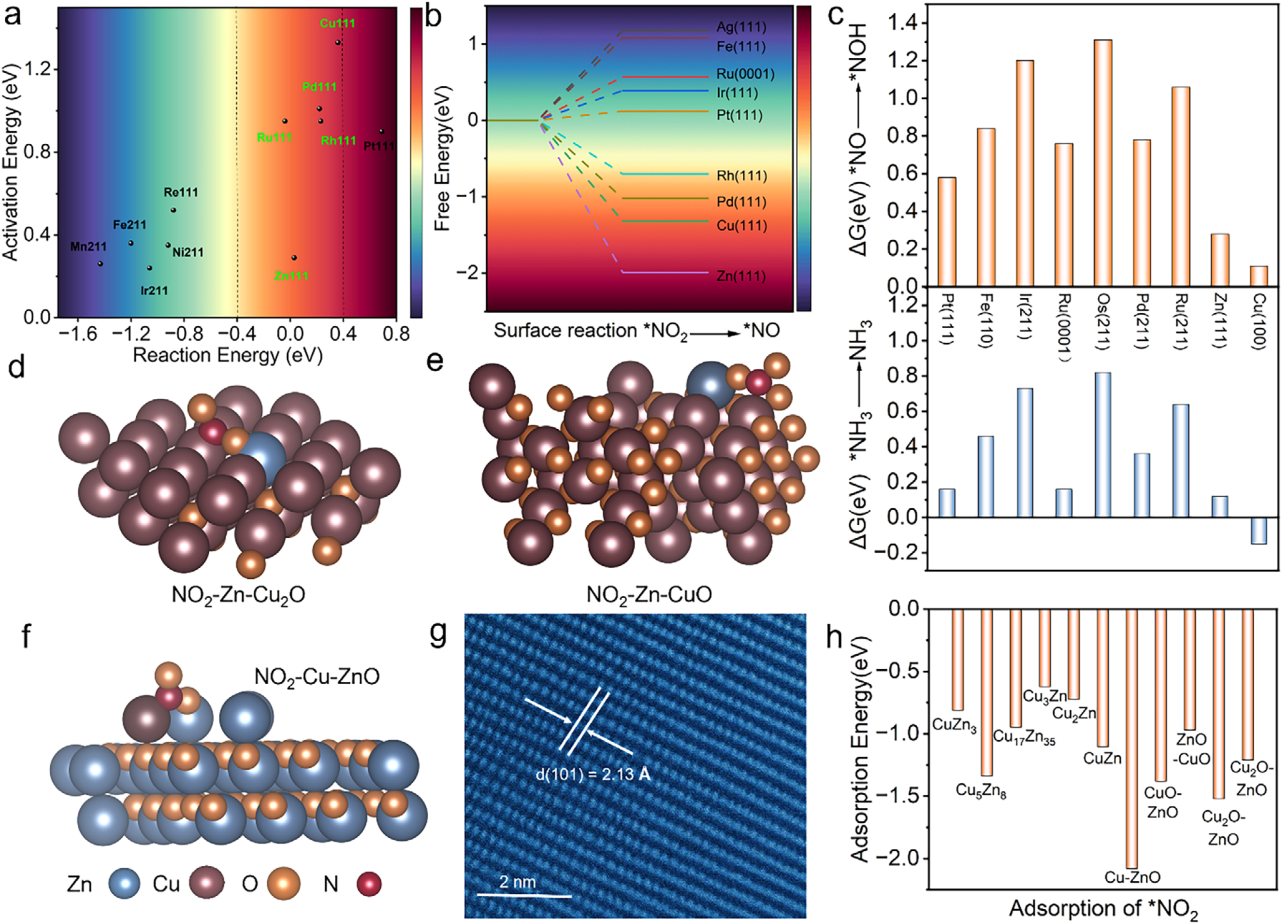
The energy and structures of computation‐guided catalysts. a) the reaction energy and activation energy of H_2_O across different metal surfaces; b) the free energy conversion from NO_2_^*^ species to NO^*^ species across various metal surfaces; c) the free energy of NH_3_ desorption and ^*^NO to ^*^NOH across different metal surfaces; d–f) structure of Zn‐Cu_2_O, Zn‐CuO and Cu‐ZnO with NO_2_^*^ species; g) HAADF‐STEM image of the obtained Cu‐ZnO; h) The adsorption energy of NO_2_^*^ species across various Cu‐Zn alloys, Zn‐Cu_2_O, Zn‐CuO and Cu‐ZnO.

The NO_2_RR process involves the chemical activation of NO_2_^−^ followed by subsequent hydrogenation steps. Accordingly, we performed DFT calculations focused on key steps including water activation, ^*^H supply, ^*^NO_2_ deoxygenation, ^*^NO hydrogenation, and ^*^NH_3_ desorption. Our study employs a structured three‐stage screening approach, starting with pure metals to establish a baseline before advancing to more complex bimetallic systems. This methodical progression ensures a logical and efficient pathway to identify optimal catalysts. we first examined the activation and reaction energies for water dissociation on various metal surfaces (**Figure** **1**a; Table S1, Supporting Information). The reaction free energy is critical; a high activation energy impedes facile water dissociation, while an overly negative reaction energy indicates strong hydrogen binding and hinders subsequent proton‐coupled steps due to competition from the hydrogen evolution reaction (HER). Therefore, for promising metals in water activation, we select those with reaction energies between −0.4 and 0.4 eV. This range suggests suitability for proton‐coupled processes within the NO_2_RR. The metals Cu, Pd, Ru, Rh, and Zn fall within this suitable range.

Subsequently, we analyze the reaction free energy for the conversion of NO_2_^*^ to NO^*^ across different metal surfaces (Figure 1b). A more negative free energy indicates greater thermodynamic favorability for this deoxygenation step. Zn(111) exhibits the lowest free energy at −1.99 eV, followed by Cu(111) at −1.32 eV. In contrast, Ag(111) and Fe(111) show high positive free energies, indicating thermodynamic unfavourability for this reaction. Based on these results, Cu, Rh, Zn, and Pd emerge as promising catalysts for promoting the ^*^NO_2_ to ^*^NO deoxygenation. Furthermore, the hydrogenation of ^*^NO to ^*^NOH and the desorption of ^*^NH_3_ are critical steps in the NO_2_RR pathway. Figure 1c compares the reaction energies for these processes across metal surfaces. On Cu (111), ΔG for the initial hydrogenation (^*^NO → ^*^NOH) is 0.11 eV, and the NH_3_ desorption requires −0.15 eV. The corresponding ^*^NO → ^*^NOH step on Zn (111) has a ΔG of 0.28 eV. Thus, Cu and Zn metals are selected as candidates for NO_2_RR. Considering the combined energetics of deoxygenation, hydrogenation, and product desorption steps, both Cu and Zn demonstrate favorable characteristics for the overall NO_2_RR process. Besides, metal oxides containing oxygen vacancies can enhance NO_2_RR performance.^[^ ^30^ ^]^ We therefore compare the adsorption energy of ^*^NO_2_ species across several dual‐site systems (Figure 1h), including Cu‐Zn alloys and ZnO‐Cu_2_O, Cu_2_O‐ZnO, ZnO‐CuO, CuO‐ZnO heterostructures (Figure S1, Supporting Information), Zn‐Cu_2_O (Figure 1d), Zn‐CuO (Figure 1e), and Cu‐ZnO (Figure 1f). It's worth noting that the adsorption of ^*^NO_2_ species on Cu‐ZnO was energetically favorable with a bridge configuration with oxygen vacancies in a minimum adsorption energy of −2.08 eV, compared with Cu, Zn, or oxygen vacancy sites (Figure S2, Supporting Information). Therefore, we identified Cu‐doped ZnO as the superior dual‐site catalyst.

To experimentally validate our theoretical findings, we synthesized Cu‐ZnO catalysts through a co‐precipitation method (detailed procedure provided in the Supporting Information). The catalyst structure was comprehensively characterized. The scanning electron microscopy (SEM) analysis revealed a predominantly plate‐like morphology (Figure S3, Supporting Information) and uniform element distribution (Figure S4, Supporting Information). High‐angle annular dark‐field scanning transmission electron microscopy (HAADF‐STEM) images (Figure 1g; Figure S5, Supporting Information) showed lattice fringes with a measured d‐spacing of 2.13Å, consistent with the (101) facet of Cu‐ZnO catalysts.

The powder X‐ray diffraction (PXRD) patterns (Figure S6, Supporting Information) confirmed the hexagonal structure of ZnO for both Cu‐doped ZnO and pristine ZnO reference, showing excellent agreement with standard reference patterns. Raman spectroscopy (Figure S7, Supporting Information) revealed a characteristic peak at around 584 cm^−1^, which is assigned to the *E*_1_^LO^ mode and strongly associated with the oxygen vacancies. Furthermore, the Tauc plot derived from UV–vis spectroscopy (Figure S8, Supporting Information) displayed a notably broad and curved absorption tail, indicating a higher concentration of structure defects within the materials.

**Figure 2:**
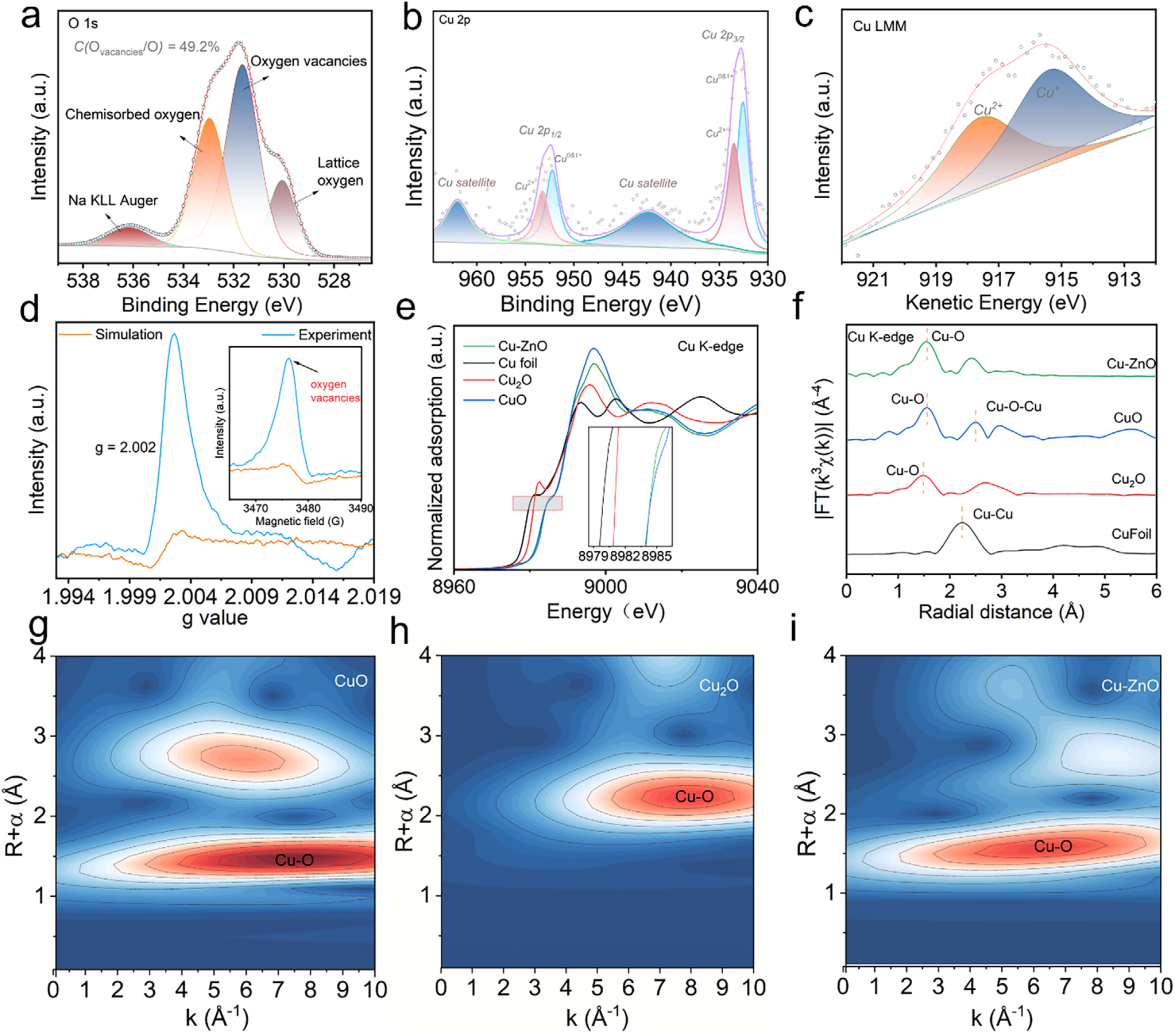
The characterization of Cu‐ZnO catalysts. a) High‐resolution XPS O 1s spectra of Cu‐ZnO; b) High‐resolution XPS Cu 2p spectra of Cu‐ZnO; c) High‐resolution XPS Cu LMM spectra of Cu‐ZnO; d) The EPR spectra of Cu‐ZnO with oxygen vacancies; e) Cu K‐edge XANES (inset is the enlarged curve); f) FT‐EXAFS spectra of Cu‐ZnO, Cu_2_O, CuO, and Cu foil; g–i) WT‐EXAFS plots of Cu‐ZnO, CuO, and Cu_2_O.

Then we used X‐ray photoelectron spectroscopy (XPS) analysis to probe the oxidation states of Cu in the synthesized materials. Deconvolution of the O 1s spectrum in **Figure** **2**a reveals three characteristic peaks. The peak at the lowest binding energy (530 eV) is attributed to lattice oxygen within the ZnO structure. The middle peak at 531.5 eV (constituting 49.2% of total oxygen species) corresponds to oxygen vacancies (O_vac_) in the ZnO lattice, which aligns well with the Raman spectrum. The higher binding energy peak at 533 eV corresponds to chemisorbed oxygen species. Analysis of the Cu 2p (Figure 2b) and Cu LMM spectra (Figure 2c) confirmed the presence of both Cu^2+^ and Cu^+^ species. Furthermore, the XPS identification of O_vac_ could facilitate the formation of low‐valent Cu^+^ species. This finding is reinforced by electron paramagnetic resonance (EPR) spectroscopy (Figure 2d), where a sharp resonance at g = 2.002 signifies localized electrons trapped at oxygen‐vacancy sites. The confirmed presence of O_vac_ directly correlates with the enhanced carrier density measured in the Cu‐ZnO catalysts.

X‐ray absorption near‐edge structure (XANES) spectroscopy was employed to probe the chemical state and local coordination environment of Cu atom in the Cu‐ZnO catalyst. The Cu K‐edge XANES spectra in Figure 2e revealed absorption edges intermediate between CuO (Cu^2+^) and Cu_2_O (Cu⁺), indicating a mixed‐valence state of Cu^2+^ and Cu⁺ species, consistent with above XPS analysis. In addition, the Fourier‐transform extended X‐ray absorption fine structure (FT‐EXAFS) spectra (Figure 2f) apparently exhibited a dominant peak at 1.47 Å due to the Cu−O scattering paths other than 2.24 Å for the Cu─Cu bond. Furthermore, the EXAFS fitting yielded a coordination number of 3.8 for Cu‐ZnO (Table S2, Supporting Information). The wavelet transforms (WT)‐EXAFS analysis (Figure 2g–i) directly visualized the Cu−O coordination environment. Combined the above XANES results with XPS spectra, we could conclude that Cu was doped on ZnO by Cu─O bond.

**Figure 3:**
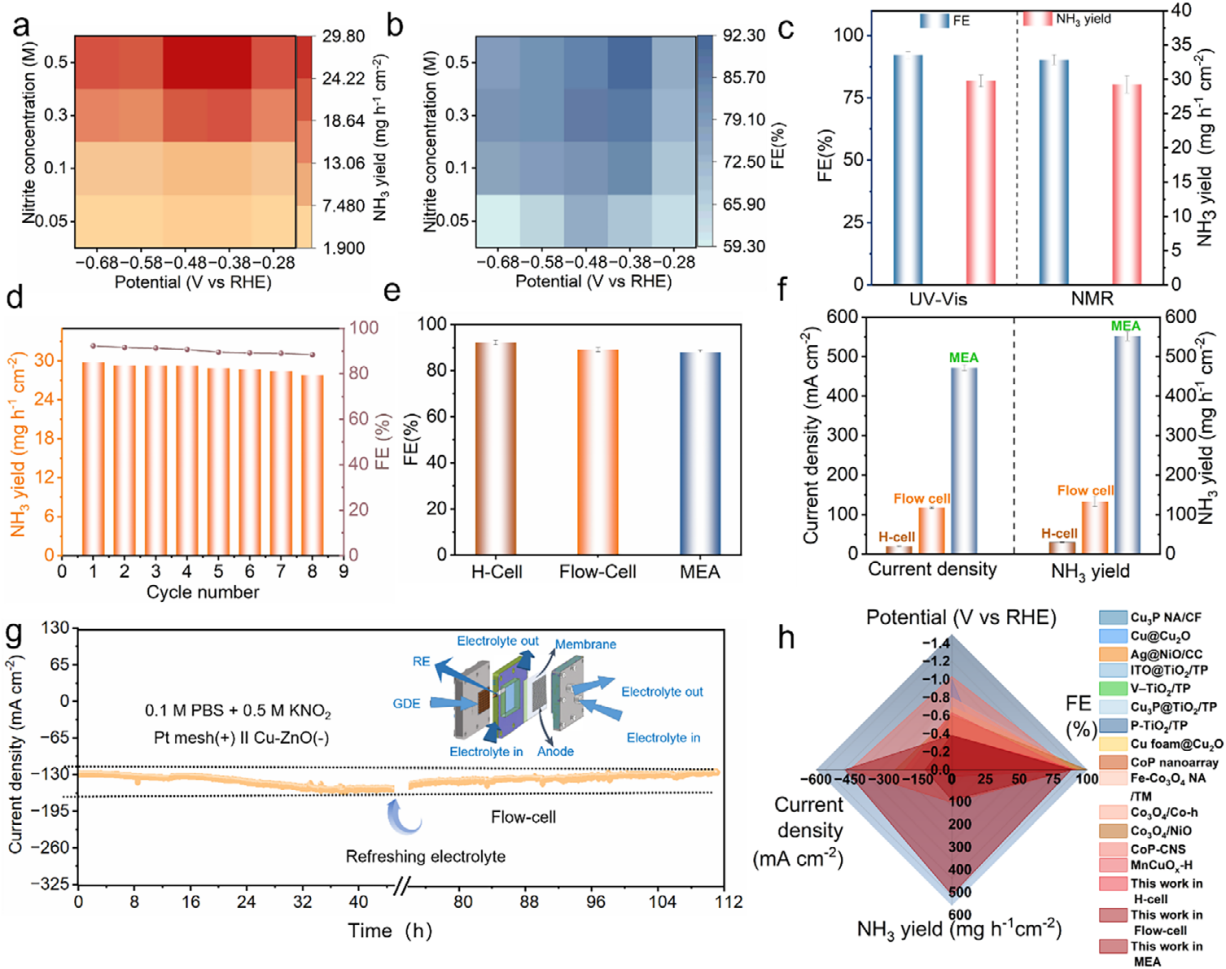
NO_2_RR performance. a,b) NH_3_ yield, and FE evaluated at various applied potentials and different concentrations; c) The NH_3_ yield, and FE determined by NMR and IBS methods at −0.38 V versus RHE; d) The stability test of Cu‐ZnO at −0.38 V versus RHE in the H cell. Each cycle required replacement of the electrolyte (0.1 m PBS containing 0.5 m KNO_2_); e,f) FE, NH_3_ yield and current density in H‐cell, flow cell and MEA; g) The stability test of Cu‐ZnO tested at −0.38 V versus RHE in the flow cell; h) The radar chart of various factors for comprehensive evaluation of NO_2_RR catalyst. (Error bars in 3c, 3e, and 3f correspond to the standard deviations of three independent measurements, and the center value for the error bars is the average of the three independent measurements.

To evaluate the NO_2_RR performances of the prepared catalysts, a sealed H‐type cell in 0.1 m PBS electrolyte containing 0.05–0.5 m KNO_2_ was set up. Linear sweep voltammetry (LSV) revealed a significant current density increase upon KNO_2_ addition, indicating high NO_2_RR activity of Cu‐ZnO (Figure S9, Supporting Information). Then, chronoamperometry coupled with indophenol blue spectrophotometric (IBS) method was utilized to quantitatively calculate the NH_3_ concentration (Figure S10, Supporting Information). Both experimental and theoretical investigations into different Cu doping amounts identified 3% as the optimal ratio for NO_2_RR, exhibiting the highest catalytic performance (Figure S11, Supporting Information) and the most favorable ^*^NO_2_ adsorption energy″ (Figures S12 and S13, Supporting Information). To evaluate the effectiveness of the Cu‐ZnO electrocatalysts, we performed control experiments against its single‐component counterparts (Cu and ZnO) and the physically mixed Cu+ZnO. Compared to the physical mixture (yield 13.92 mg h^−1^ cm^−2^, FE 83%), the Cu‐ZnO catalyst achieves a significantly higher NH_3_ yield (29.75 mg h^−1^ cm^−2^) and Faradaic Efficiency (92%), demonstrating the essential role of Cu‐Zn bridge sites in achieving high catalytic activity (Figure S14, Supporting Information). **Figure** **3**a,b displayed the potential‐dependent NH_3_ yield and FE for Cu‐ZnO from −0.28 to −0.68 V in electrolyte with varying KNO_2_ concentrations (0.05–0.5 m). Both NH_3_ yield and FE exhibit volcano‐shaped trends, peaking at −0.38 V with a maximum FE of 92.2% and NH_3_ yield of 29.75 mg h^−1^ cm^−2^. The initial increasement from −0.28 to −0.38 V versus RHE is ascribed to accelerated NO_2_RR reaction kinetics and efficient ^*^H utilization. Beyond −0.38 V, gradually increased HER resulted in reduced NH_3_ yield and FE. To further determine the optimal KNO_2_ concentration, NO_2_RR performance was systematically evaluated across concentrations from 0.05 to 0.5 m.

Increasing KNO_2_ concentration could significantly enhance both NH_3_ yield and FE due to the improved mass transport and increased NO_2_^−^ availability at active sites. It could be found that the FE and NH_3_ yield confirmed by ^1^H nuclear magnetic resonance (NMR) is well aligned with the data measured by IBS (Figure 3c; Figure S15, Supporting Information), validating the accuracy and robustness of both methods in this work. In addition, side product analysis revealed trace hydroxylamine (NH_2_OH) but no detectable hydrazine (N_2_H_4_). The high NH_3_ selectivity was verified by analyzing all nitrogenous byproducts via online DEMS and UV–vis, yielding a nitrogen balance of ≈98% (Figures S16–S19, Supporting Information), which validates the reliability of our Faradaic efficiency calculations. In addition to high NH_3_ yield and FE, the catalyst's stability was also crucial for practical applications. We then evaluate the stability of our Cu‐ZnO catalysts through a 480 min durability test in an H cell at −0.38 V, with NH_3_ yield and FE remaining 93.4% and 95.8% respectively (Figure 3d). These experimental results strongly validate our DFT predictions.

To enhance mass transfer, we evaluated NO_2_RR performance in flow cell and membrane electrode assembly (MEA) electrolyzers. The Cu‐ZnO catalyst achieved an NH_3_ yield of 133.45 mg h^−1^ cm^−2^ and a current density of 117.6 mA cm^−2^ in the flow cell, which is 4.5 times over the H‐cell configuration. This enhancement stems from a shorter distance between electrodes and lower internal resistance, facilitating improved mass transfer and higher current density (Figure 3e,f). Further transforming to an MEA electrolyzer eliminated reference electrode constraints, yielding even lower internal resistance and exceptional performance: 552.16 mg h^−1^ cm^−2^ NH_3_ production and a current density of 480.2 mA cm^−2^ (Figure S20, Supporting Information), representing an additional 4.1 times increase over the flow cell. Notably, the FE remains nearly constant across all reactors, confirming sustained high selectivity. The catalyst system demonstrated remarkable stability during over 96 h continuous flow cell operation at −0.38 V versus RHE, without significant degradation (Figure 3g). Notably, Cu‐ZnO exhibits outstanding electrocatalytic properties (i.e., NH_3_ yield, FE, current density, and potential), being superior to many reported catalysts (Figure 3h; Table S3, Supporting Information).

**Figure 4:**
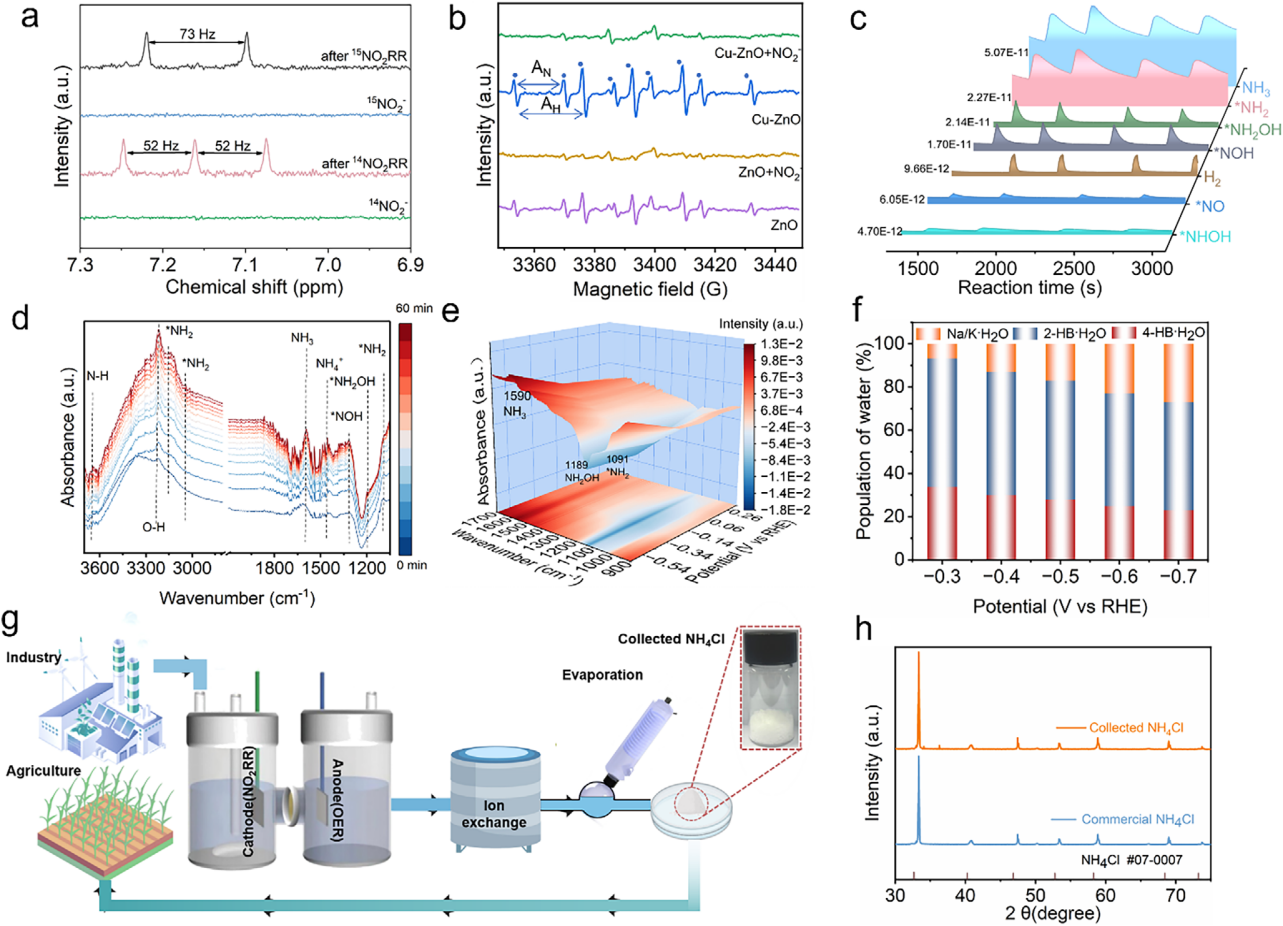
NO_2_RR mechanism and reaction pathway. a) Isotopic labeling with ^15^N as the nitrogen source; b) EPR spectra of electrocatalysts under different conditions; c) In situ DEMS results of Cu‐ZnO; d) Time‐dependent in situ ATR‐SEIRAS spectra; e) In situ ATR‐IRAS measurement under different applied potentials; f) The relative proportions of three types of water at different potential; g) Schematic of the synthesis process from nitrite‐containing influent to NH_4_Cl (s); h) XRD pattern of synthesized and commercial NH_4_Cl solid products.

To further investigate the origin of the generated NH_3_, a ^15^N isotope labeling experiment was conducted using ^1^H NMR spectroscopy with either K^15^NO_2_ or K^14^NO_2_ as the nitrogen source (**Figure** **4**a). When K^14^NO_2_ was used, the ^1^H NMR spectrum displayed a characteristic triplet signal for ^14^NH_4_^+^ with a coupling constant of 52 Hz. In contrast, when K^15^NO_2_ served as the nitrogen precursor, a doublet signal corresponding to ^15^NH_4_^+^ was observed, exhibiting a coupling constant of 73 Hz. A series of control experiments indicates that the nitrogen source in the NH_3_ product only originates from NO_2_^−^ (Figure S21, Supporting Information). Carbon paper itself shows negligible NO_2_RR activity, demonstrating that NH_3_ is mainly produced on Cu‐ZnO. The above results confirm that the produced NH_3_ originates from the electroreduction of NO_2_^−^, rather than from nitrogen‐containing impurities or species present in the catalyst.

Given the critical role of active H^*^ in the NO_2_RR process, we investigated the H^*^ production capability of our catalyst through electron paramagnetic resonance (EPR) spectroscopy. The DMPO‐H signal exhibited an intensity ratio of 1:1:2:1:2:1:2:1:1, which aligns with the calculated hyperfine coupling constants of A_N_ = 16.4 G and A_H_ = 22.6 G (Figure 4b). It could also be observed that Cu‐ZnO exhibits remarkable H^*^ generation capacity during electrolysis without nitrite. Upon nitrite introduction, the disappearance of active H^*^ signals confirmed rapid H^*^ consumption for the NO_2_RR hydrogenation process. Comparative analysis demonstrates that the H^*^ generation of Cu‐ZnO is significantly higher than that of ZnO. These compelling findings substantiate the essential role of doped Cu atoms in regulating H^*^ formation and utilization. To explore the effectiveness of water dissociation in governing the reaction kinetics and the origin of H^*^ in the catalytic process, we conducted H_2_O/D_2_O isotope experiments. The observed KIE value of 3.2 from Tafel analysis identifies water dissociation as the rate‐limiting step, verifying water as the exclusive source of reactive hydrogen (H^*^) (Figure S22, Supporting Information). The in situ EPR monitoring (Figure S23, Supporting Information) reveals decay of H^*^ signals during NO_2_RR, confirming continuous consumption in proton‐coupled electron transfer steps. This time‐dependent signal decrease directly reflects the reaction kinetics of proton transfer kinetics in NO_2_RR. Furthermore, the XRD, XPS, and TEM after cycle test revealed unchanged morphology and structure (Figures S24,S25, and S27, Supporting Information), demonstrating the excellent structural robustness of Cu‐ZnO. The Cu 2p spectrum shows a slight shift to higher binding energy, indicating a slightly dynamic Cu⁺/Cu^2^⁺ interconversion. Simultaneously, the Zn 2p spectrum exhibits subtle peak sharpening, reflecting Zn's participation in electron transfer with Cu (Figures S24–S27, Supporting Information).

To gain a deeper understanding of the reaction pathways during NO_2_RR, the intermediates and products generated were captured via DEMS. When using KNO_2_ as the reactant, a prominent signal at ^*^m/z ^*^17 and a weaker peak at ^*^m/z ^*^2 was detected, corresponding to the primary product NH_3_ and the by‐product H_2_, respectively (Figure 4c). Furthermore, the signal of HNO^*^/NOH^*^, NH_2_OH^*^, and NH_2_^*^ signals suggested the following potential reaction pathway: NO_2_^−^ → NO_2_^*^ → NO^*^→HNO^*^/NOH^*^ → NHOH^*^→NH_2_OH^*^ → NH_2_^*^ → NH_3_^*^ → NH_3_. To further monitor and identify possible intermediates, we employed in situ attenuated total reflection‐surface enhanced infrared absorption spectroscopy (ATR‐IRAS). The reaction was conducted in 0.1 m PBS containing 0.5 m KNO_2_ solution at −0.38 V versus RHE for 60 min. As shown in Figure 4d, peaks at 1590 cm^−1^ were attributed to the H‐N‐H bending of NH_3_. A gradually increasing peak at 1456 cm^−1^ indicated the accumulation of NH_4_^+^.^[^ ^31^ ^]^ The peaks at 1091, 3041, 3153 cm^−1^ corresponded to the vibration of ^*^NH_2_.^[^ ^32^ ^]^ It was noteworthy that the presence of the N‐O stretching vibration of ^*^NH_2_OH species located at 1189 cm^−1^ was observed,^[^ ^33^ ^]^ which is a key intermediate captured by DEMS as well. There was a broad overlapped band ranging from 3100 to 3700 cm^−1^, where the bands at 3216 and 3644 cm^−1^ were attributed to O−H and N−H, respectively, representing the consumption of protons from water and the process of hydrogenation. It's noteworthy that the N‐O stretching vibration of ^*^NOH species located at 1313 cm^−1^ were clearly observed. in situ ATR‐IRAS presented similar peaks when measured at different voltages (Figure 4e; Figure S28, Supporting Information). In combination the DEMS and FTIR results, the reaction pathway was identified as follows: NO_2_^−^ → NO_2_^*^ → NO^*^→NOH^*^ → NHOH^*^→NH_2_OH^*^ → NH_2_^*^ → NH_3_^*^ → NH_3_.

Interfacial water, comprising both hydrogen‐bonded and metal‐ion‐coordinated water, serves as the primary source of H^*^ during NO_2_RR.^[^ ^34^ ^]^ To characterize these interfacial water structures, we performed Gaussian deconvolution of the broad O−H stretching bands (2800–3800 cm^−1^) in ART‐FTIR spectra. This region could be resolved into three distinct peaks (Figure 4f; Figure S29, Supporting Information), corresponding to 4‐coordinated hydrogen‐bonded water (4‐HB⋅H_2_O), 2‐coordinated hydrogen‐bonded water (2‐HB⋅H_2_O), and water coordinated with Na^+^/K^+^ ions (Na/K⋅H_2_O), arranged in order of increasing wavenumber.^[^ ^35^ ^]^ Notably, as the applied potential decreased, the proportion of Na/K⋅H_2_O, representing weaker hydrogen bonding, increased from 6.76% to 27.03%. The potential‐dependent increase demonstrates that Na/K⋅H_2_O possesses enhanced water dissociation. To validate the catalyst's applicability in real environments, we evaluated its performance in a simulated wastewater containing a range of nitrite concentrations (0.05–0.5 m) and common inorganic ions (e.g., HPO_4_^2−^/H_2_PO_4_^−^). As shown in Figure S30 (Supporting Information), Cu‐ZnO maintained considerable Faradaic efficiency across these different concentrations at different applied potentials, confirming robustness and functional stability in such systems (Figure S30, Supporting Information). Besides, the electrochemically active surface area (ECSA) via double‐layer capacitance further verified the high intrinsic activity of Cu‐ZnO (Figure S31, Supporting Information). Furthermore, the Cu‐ZnO catalyst maintained stable performance throughout a 40 h operation in the MEA, validating its durability in harsh operational environments (Figure S32, Supporting Information). We have also achieved high energy efficiency (EE ≈ 74.3%) and superior specific energy consumption (SEC = 13.9 kWh kg^−1^ NH_3_) to reflect the process's practical viability.

To demonstrate the practical application potential of our Cu‐ZnO electrocatalytic system, the high‐purity ammonia products were continuously collected from a set of tailor‐made devices (Figure 4g). After 24 h of electrolysis at −0.38 V versus RHE, a hydrochloric acid adsorption unit is introduced for ammonia capture. Subsequent purification through HPO_4_^−^ and Cl^−^ ion exchange and rotary evaporation to yield concentrated ammonium chloride solutions, which were ultimately converted into high‐purity NH_4_Cl powder. The powder product is highly consistent with standard NH_4_Cl (JCPDS NO. 07‐0007), as confirmed by the XRD pattern (Figure 4h). Thus, this high‐grade NH_4_Cl demonstrates significant commercial potential as a precursor material for fertilizer, textile, and pharmaceutical, establishing a practical pathway for sustainable ammonia utilization.

**Figure 5:**
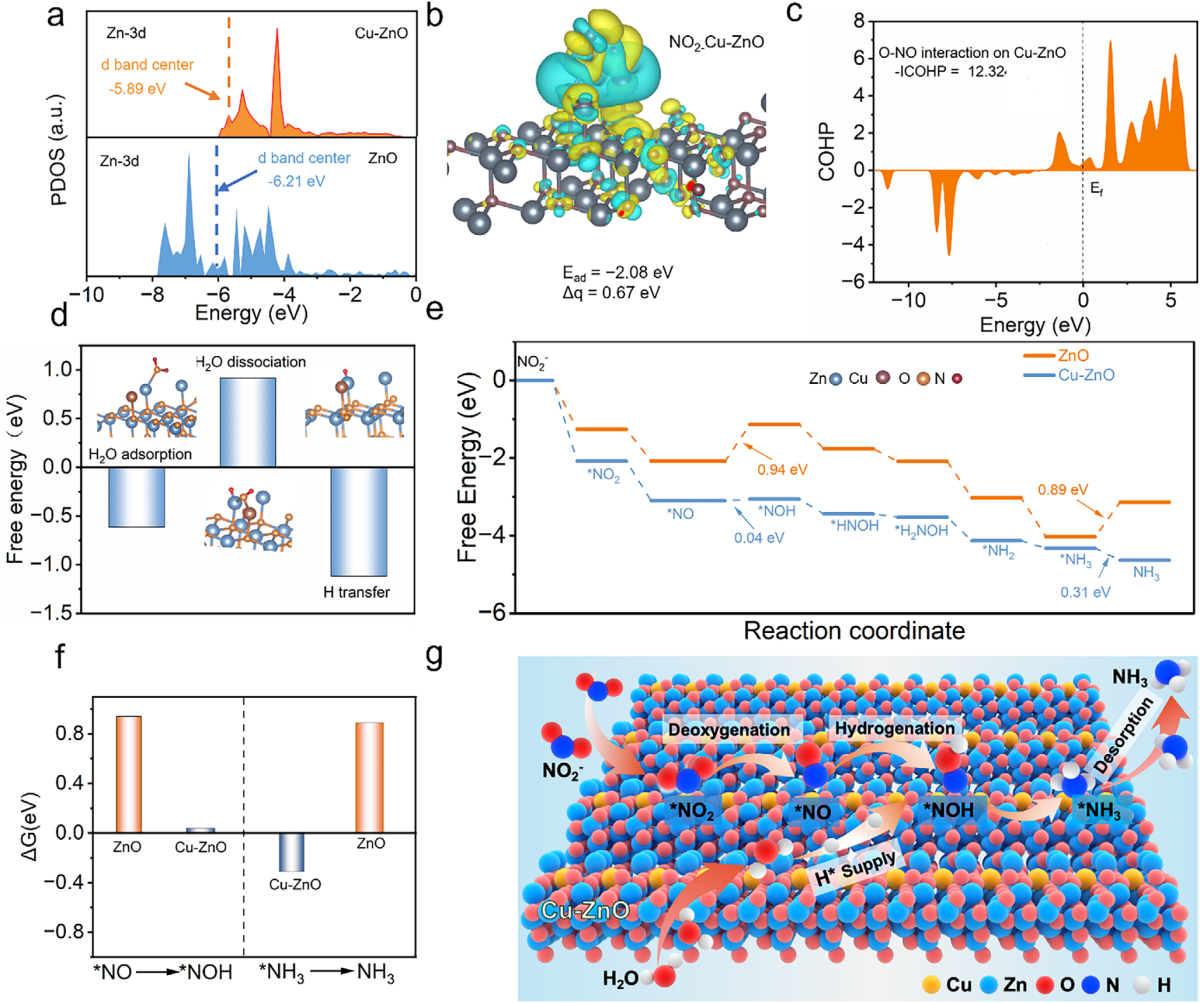
Theoretical calculations of NO_2_RR over catalysts. a) The projected density of states (PDOS) and d band center of Cu‐ZnO and ZnO; b) Differential charge density of Cu‐ZnO with NO_2_^*^ adsorption, yellow and cyan denote charge accumulation and depletion regions, respectively; c) Energy barriers and water interaction models, the energy barriers for water adsorption, dissociation, and H production; d) The Crystal Orbital Hamilton Populations (COHP) for O─NO bonds n Cu‐ZnO; e) Free energies of the NO_2_RR reaction pathway on Cu‐ZnO; f) The energy of ^*^NO to ^*^NOH and ^*^NH_3_ to NH_3_ in ZnO and Cu‐ZnO; g) The mechanism of NO_2_RR process over Cu‐ZnO.

To investigate the electronic structure evolution of the catalyst surface during NO_2_RR, theoretical calculations were performed. The projected density of states (PDOS) of Cu‐ZnO and ZnO revealed that Cu doping shifts the d‐band center of Zn's 3d orbitals upward from −6.21 to −5.89 eV (**Figure** **5**a). This shift reduces the occupation of antibonding orbitals, favoring the binding of the adsorbed ^*^NO_2_ intermediate and facilitating its deoxygenation to ^*^NO. This provides a valid explanation for the strengthening of ^*^NO_2_ adsorption. The electronic structures of Cu‐ZnO with absorbed NO_2_^−^ were also calculated to observe the electron transfer. As shown in Figure 5b, the noticeable electron transfer from Zn to NO_2_^−^ was clearly observed from the charge density difference analysis. Bader charge analysis quantitatively confirms this interaction, showing a decrease in Zn electron count from 11.02 to 10.35 and a corresponding electron accumulation of 0.67 e^−^ on NO_2_^−^. The PDOS after NO_2_^−^ adsorption shows the overlap between Zn‐3d and N‐2p orbitals, ensuring the strong interaction between our catalysts with NO_2_^−^, which was significant to further NO_2_RR reaction (Figure S33, Supporting Information).

Crystal Orbital Hamilton populations (COHP) analysis of the N─O bonds in the Cu‐ZnO‐NO_2_ system reveals antibonding character, strengthening activation of the ^*^NO_2_ species. The ‐ICOHP value for Cu‐ZnO‐NO_2_ was 12.32, which is lower than that of ZnO‐NO_2_ (14.36), indicating facilitated ^*^NO_2_ activation for subsequent hydrogenation (Figure 5c; Figure S34, Supporting Information). This is consistent with the enhanced adsorption of NO_2_^−^ after Cu doping. Theoretical calculations further probe the H^*^ generation and utilization, focusing on the H_2_O dissociation pathway (Figure 5d). Adsorption calculations show that of Zn function as active sites for water dissociation, providing an efficient H^*^ supply. The Cu plays a key role by stabilizing the ^*^OH intermediate in a bridge configuration between Cu and Zn atoms, thereby modulating the H_2_O dissociation process. The proximity of Zn sites allows for immediate utilization of the produced H^*^ for hydrogenation steps. This efficient utilization prevents the accumulation of active H^*^ and suppresses the competing HER.

The reaction pathway from nitrite to ammonia is further confirmed via theoretical calculations (Figure 5e; Figure S35, Supporting Information). Adsorption of NO_2_^−^ onto Cu‐ZnO forms the ^*^NO_2_ intermediate in a bridge configuration (N and O bound to Cu and Zn atoms), with a favorable Gibbs free energy change of −2.08 eV. Subsequent proton capture form water dissociation converts ^*^NO_2_ to ^*^NO species, which has a decrease in Gibbs free energy by −1.02 eV. ^*^NO is a key intermediate that influence the product selectivity. Subsequently, the NO_2_RR process follows the following pathway: ^*^NO→^*^NOH→^*^NHOH→^*^NH_2_OH→^*^NH_2_→^*^NH_3_→NH_3_. In conclusion, the above simulated results are highly consistent with the experimental conclusions from in situ DEMS and ATR‐IRAS. To elucidate the influence of proton‐coupled electron transfer (PCET) on the reaction pathway and selectivity, the DFT calculations, in situ EPR, and H_2_O/D_2_O isotope experiments have been sufficiently conducted to validate PCET's regulatory role. These findings are complemented by product analyses, which further reflect its link to selectivity under neutral conditions.

Reaction kinetics and theoretical analyses reveal that the high nitrite‐to‐ammonia activity arises from favorable hydrogenation kinetics and desorption thermodynamics. The energy barrier for ^*^NOH formation is significantly reduced to 0.04 eV on Cu‐ZnO, compared to 0.94 eV on pristine ZnO (Figure 5f; Figure S36, Supporting Information), highlighting Cu doping's critical role in facilitating ^*^NO hydrogenation. Furthermore, NH_3_ desorption is thermodynamically spontaneous on Cu‐ZnO (ΔG = −0.31 eV), whereas it remains endergonic on ZnO (ΔG = +0.89 eV). Based on these findings, a novel catalytic mechanism is proposed for NO_2_RR over Cu‐ZnO (Figure 5g). The Zn and Cu sites synergistically serve as dual adsorption and activation centers, where NO_2_^−^ adsorbs in a bridge configuration for primary activation. Adjacent Zn sites then facilitate H_2_O dissociation, while Cu doping modulates the water dissociation kinetics to enable efficient generation of active H^*^ species. This optimized H^*^ supply drives rapid proton‐coupled electron transfer steps during NO_2_RR process.

## 3 Conclusion

In summary, we established a theory‐guided, experimentally validated approach for efficient nitrite‐to‐ammonia conversion, exemplified by Cu–ZnO dual‐site catalysts. First‐principles calculations identified Cu doping as a means to upshift the Zn d‐band center and reduce antibonding orbital occupation, thereby strengthening ^*^NO_2_ adsorption and accelerating deoxygenation to ^*^NO. Simultaneously, ZnO sites enhance water dissociation, creating a proton‐rich interfacial microenvironment that drives rapid ^*^NO hydrogenation. This dual‐site synergy precisely tailors nitrite activation and hydrogenation kinetics, suppressing competing hydrogen evolution and maximizing NH_3_ yield and Faradaic efficiency. Guided by computation and confirmed through DEMS, ATR‐IRAS, and multi‐reactor testing (H‐cell, flow cell, MEA), the Cu–ZnO catalysts achieved record NO_2_RR performance with sustained stability. This mechanism‐to‐device workflow replaces trial‐and‐error with predictive catalyst design, offering a generalizable strategy for developing advanced electrocatalysts and scalable reactor systems for targeted electrochemical transformations.

## 4 Experimental Section

Details on sample preparation, characterizations, and electrochemical measurements can be found in the supporting information.

## Conflict of Interest

The authors declare no conflict of interest.

## Supporting information



Supporting Information

## Data Availability

The data that support the findings of this study are available from the corresponding author upon reasonable request.
