# Supplementary material for: Computation‐Guided Dual‐Site Electrocatalysts for Record‐Performance Nitrite‐to‐Ammonia Conversion
Source: Adv Sci (Weinh). 2025 Dec 17;13(13):e20683. doi: 10.1002/advs.202520683 (PMC12955985; doi:10.1002/advs.202520683)
Supplement: Supplementary file 1 — Supporting Information [file ADVS-13-e20683-s001.docx]

***Supporting Information***

**Computation-Guided Dual-Site Electrocatalysts for Record-Performance Nitrite-to-Ammonia Conversion**

*Hui Zhang^1^, Haiyan Duan^1*^, Donglin Han^1^, Zhenlin Wang^1^, Xingchi Li^1^, Dengchao Peng^1^, Lupeng Han^1^, Tianting Pang^1^, Evangelina Pensa^2^, Wenqiang Qu^4^, Yongjie Shen^5^, Haotian Wang^3^, Wei Ren^3*^, Ming Xie^6^, Emiliano Cortés^2*^, Dengsong Zhang^1*^*

^1^Innovation Institute of Carbon Neutrality, International Joint Laboratory of Catalytic Chemistry, State Key Laboratory of Advanced Special Steel, Department of Chemistry, College of Sciences, Shanghai University, Shanghai 200444, China.

^2^Nanoinstitute Munich, Faculty of Physics, Ludwig-Maximilians-Universität (LMU), Munich 80539, Germany.

^3^Materials Genome Institute, Qianweichang College, Shanghai University, Shanghai 200444, People’s Republic of China.

^4^ Department of Chemistry, University of Toronto, 80 St. George Street, Toronto, ON M5S 3H6, Canada.

^5^ Institute for Chemical Reaction Design and Discovery (WPI-ICReDD), Hokkaido University, Sapporo 001-0021, Japan.

^6^ Department of Chemical Engineering, University of Bath, Bath BA2 7AY, U.K.

* Corresponding Authors: [haiyanduan@shu.edu.cn](mailto:haiyanduan@shu.edu.cn); [renwei@shu.edu.cn;](mailto:renwei@shu.edu.cn;) [Emiliano.Cortes@lmu.de](mailto:Emiliano.Cortes@lmu.de); dszhang@shu.edu.cn.

**Contents**

1. Materials and Chemicals
2. Experimental Section
3. Figures and Tables

Figure S1. Six Cu-Zn alloys and four heterostructures with different crystal structures (blue and red represent Zn and Cu atoms, respectively).

Figure S2. The structures and adsorption energy of *NO_2_ across varying sites of Cu-ZnO.

Figure S3. SEM image of Cu-ZnO.

Figure S4. EDS mapping of various elements for Cu-ZnO.

Figure S5. TEM image of Cu-ZnO.

Figure S6. XRD pattern of Cu-ZnO and pristine ZnO.

Figure S7. Raman spectra of Cu-ZnO.

Figure S8. The Tauc plot illustrates the optical band gap of Cu-ZnO.

Figure S9. The LSV curve of Cu-ZnO catalyst.

Figure S10. UV-Vis absorption spectra of NH_4_Cl standard solutions and corresponding calibration curve.

Figure S11. FE and NH_3_ yield of Cu-ZnO with varying Cu doping amounts, bar charts represent the NH_3_ yield, while scatter plots represent the FE.

Figure S12. The adsorption energy of *NO_2_ in Cu-ZnO with varying Cu doping amounts.

Figure S13. The structures of Cu-ZnO with absorbed *NO_2_ with varying Cu doping amounts.

Figure S14. (a) FE and (b) NH_3_ yield of Cu-ZnO, Cu, physically mixed Cu+ZnO, and ZnO.

Figure S15. ^1^H-NMR spectra of NH_4_^+^ standard solutions and corresponding calibration curve.

Figure S16. UV-Vis absorption spectra of NH_2_OH standard solution.

Figure S17. UV-Vis absorption spectra of N_2_H_4_ standard solution.

Figure S18. FE and NH_3_ yield of Cu-ZnO and ZnO.

Figure S19. Online differential electrochemical mass spectrometry (DEMS) spectra over Cu-ZnO.

Figure S20. The i-t curve for NO_2_RR in the MEA reactor.

Figure S21. The NH_3_ yield for NO_2_RR in the H-cell, O/C stands for open circuit voltage, and CP stands for carbon paper without catalysts.

Figure S22. (a) The polarization curves of Cu-ZnO in H_2_O and D_2_O, (b) Tafel plots derived from the. polarization curves in a.

Figure S23. The in situ EPR signals for NO_2_RR.

Figure S24. The XRD patterns of Cu-ZnO catalyst coated on the gas diffusion layer (GDL) during the. electrochemical test.

Figure S25. The Cu 2p XPS pattern of Cu-ZnO during the electrochemical test.

Figure S26. The Zn 2p XPS pattern of Cu-ZnO during the electrochemical test.

Figure S27. The TEM pattern of Cu-ZnO after the electrochemical test.

Figure S28. The in-situ ATR-IRAS measurement under different applied potentials.

Figure S29. The corresponding Gaussian-fitted peaks reveal the three O-H stretching modes of interfacial water on Cu-ZnO.

Figure S30. FE of Cu-ZnO in a simulated wastewater system containing representative concentrations of nitrite (0.05-0.5 M KNO_2_) and common inorganic ions (e.g., HPO_4_^2⁻^/H_2_PO_4_^⁻^).

Figure S31. (a) The CV curves of Cu-ZnO at various scan rates, (b) corresponding calibration curve used for the calculation of C_dl_.

Figure S32. The stability test of Cu-ZnO in the MEA configuration at -1.6 V (electrolyzer cell voltage).

Figure S33. The projected density of states (PDOS) after NO_2_^−^ adsorption.

Figure S34. The COHP of the O-NO interaction on ZnO.

Figure S35. The reaction pathway of NO_2_RR.

Figure S36. The illustration showing the easy hydrogenation and desorption of Cu-ZnO.

Table S1. The activation and reaction energy of H_2_O to *H on various metal surfaces.

Table S2. EXAFS fitting parameters at the Cu K-edge for Cu-ZnO, CuO, Cu_2_O and Cu foil.

Table S3. The comparison of NH_3_ yield and FE with reported literatures.

# 1. Materials and Reagents

All the chemicals were used without further purification. Copper nitrate hydrate (Cu(NO₃)₂·3H₂O) and Zinc nitrate (Zn (NO_3_)_2_) were purchased from Sinopharm Chemical Reagent Co., Ltd, China. Sodium hydroxide, sodium citric, (>99%), salicylic acid, sodium hypochlorite (NaClO), sodium nitro-ferricyanide dihydrate (99%), ammonium chloride NH_4_Cl (99.99%) were procured from Merck, Ltd., China. PBS buffer (10x,pH 7.2-7.4) was purchased from adamas. Ultrapure gases (argon) were purchased from YouJiaLi Liquid Helium Co., Ltd, China.

2. Experimental Section

**2.1 Synthesis of Cu-ZnO.**

The synthesis of Cu-ZnO was carried out through a co-precipitation method. Initially, 5.954 g Zn (NO_3_)_2_ and 0.2658 g Cu(NO_3_)_2_·3H_2_O were added to 50 mL of deionized water, 20 g NaOH and 18 mL of deionized water were used to prepare a NaOH solution with a molar ratio of 1:2, and the sample was dissolved in the deionized water and then NaOH solution was added drop by drop at room temperature, and the addition of NaOH solution was stopped at pH 11, and then the filtration process was carried out, and the precipitate was dried. The precipitate was cleaned with deionized water and ethanol, and then the precipitate was dried in a muffle furnace at 500 °C for 2 hours.

**2.2 Characterizations.**

X-ray diffraction (XRD) patterns were recorded on a Bruker D8 Advance diffractometer (Cu-Kα radiation, λ = 1.5406 Å, 40 kV, 40 mA) in the 2θ range of 2°-40°. The surface morphology and elemental distribution were characterized using a field emission scanning electron microscope (FE-SEM, Sigma-300; Zeiss, Germany) equipped with an energy-dispersive spectrometer (EDS), and a transmission electron microscope (TEM, JEM-2100F; JEOL, Japan) with EDS capability.

X-ray photoelectron spectroscopy (XPS) analysis was performed using a PerkinElmer PHI-5300 system with Mg-Kα excitation (1253.6 eV). The X-ray absorption near edge structure (XANES) and extended X-ray absorption fine structure (EXAFS) spectra were tested at SSRF BL17B1 of the National Facility for Protein Science in Shanghai (NFPS), Shanghai Advanced Research Institute.

**2.3 Electrochemical Measurements.**

**H-cell measurement:** Electrochemical analyses were conducted using a sealed H-type cell in 0.1 M PBS buffer solution with 0.05 M-0.5 M KNO_2_ separated by Nafion 117 membrane. Prior to testing, the Nafion membrane were first immersed in 5 wt% H₂O₂ solution at 80 °C for 1 hour, followed by 0.5 M H₂SO₄ treatment for 2 hours, and finally rinsed with deionized water for 6 hours at room temperature. The NO_2_RR experiments were carried out with a three-electrode system having a graphite rod as the counter electrode, Ag/AgCl (saturated KCl) as the reference electrode, and Cu-ZnO as the working electrode. The 5 mg catalysts, 25 μL Nafion (5%), 150 μL ultrapure water and 300 μL isopropanol were mixed and sonicated to get ink, then drop-casted onto the carbon paper (TGP-H-060 0.2 mm) through multiple coating cycles to achieve a loading of 1 mg/cm². A CHI 760E electrochemical workstation (Chenghua Inc., Shanghai, China) was employed for data acquisition. The NO_2_RR characteristics of catalyst have been explored by employing cyclic voltammetry (CV) at a scan rate of 50 mV/s, linear sweep voltammetry (LSV) within a potential range of -0.78 V to 0.32 V (vs RHE) at a scan rate of 5 mV/s without IR compensation, as well as chronoamperometry method from -0.28 V to -0.68 V (Vs RHE) lasting 1 hour. Before NO_2_RR measurements, the cathodic chamber was purged with high-purity Ar gas for 30 minutes to remove residual O_2_ and N_2_. For consistency, all recorded potentials were converted to the reversible hydrogen electrode (RHE) scale using the following Equation.

E_RHE_ = E_Ag/AgCl_+0.197+0.0591×pH (pH = 7.2 in PBS buffer)

**Flow cell measurement:** A commercial flow cell electrolyzer (model 2*2 cm-T2, Shanghai Chuxi Co., Ltd.) was utilized for continuous-flow measurements in three-electrode system, which were consisted of a Pt mesh as the counter electrode, a saturated calomel electrode (SCE) as the reference, and the catalyst-coated gas diffusion electrode (GDE, YLS-30T, 4 cm²) as the working electrode. The catalyst-coated side faced the catholyte compartment, the opposite side interfaced with the gas chamber (there is no gas feedstock in NO_2_RR). The anode and cathode compartments were separated by a Nafion 117 membrane. Before measurements, the gas chamber was purged with Ar (30 mL/min) for 30 minutes to eliminate O_2_ and N_2_, the electrolyte was recirculated via a peristaltic pump.

E_RHE_ = E_SCE_ +0.241 +0.0591×pH (pH = 7.2 in PBS buffer)

**Membrane electrode assembly (MEA) measurement:** The MEA electrolyzer is a commercial equipment (model 2*2 cm-T2, Shanghai Chuxi Co., Ltd.). The catalyst ink was drop-casted onto a gas diffusion electrode (GDE, YLS-30T, 4 cm²). The MEA electrolyzer was constituted with titanium flow fields for the anode and cathode sides, the Pt mesh as the counter electrode, and the catalyst-coated side as the working electrode. The flow channel in both anodic and cathodic side was supplied with 0.1M PBS and 0.5 M KNO_2_.

**2.4 Product Detection.**

**Determination of NH_3_:** The NH_3_ yield was determined by using the indophenol blue spectrophotometric (IBS) method and ^1^H nuclear magnetic resonance (^1^H NMR, AVANCE NEO 600 MHz, Bruker). A specific volume of cathodic electrolyte was collected after 1 hour of electrolysis and subsequently diluted to fall within the detection range. Then, 2 mL of the reagent A, 2 mL of the reagent B, and 200 μL of the reagent C, are introduced to the 2 mL diluted catholyte. The reagents A, B, and C are prepared using the following methods. Reagent A (100 mL deionized water, 4.39 g sodium citrate, 5 g salicylic acid), reagent B (100 mL deionized water, 8.9 mL 4% sodium hypochlorite), reagent C (25 mL deionized water, 0.25 g sodium nitroprusside (Na_2_[Fe(CN)_5_NO]). After being kept in the dark for approximately 1.5 hours, the UV-vis absorbance at 655 nm of the mixed solutions was then measured. To ascertain the concentration of NH_4_^+^, a calibration curve is created using a range of standard ammonium sulfate (NH_4_Cl) solutions, with concentrations varying from 0.5 to 4.0 ppm. The ammonia yield and Faradaic efficiency (FE) are calculated using the following formulas:

$$\text{ NH}_{3} \mathrm{yield} \left( m\text{g h}^{-1}\text{cm}^{-2} \right)=\frac{\left( C_{\mathrm{NH}_{3}}\times V \right)}{\left( t\times A \right)}$$

$$\mathrm{FE}\left( \% \right)=\frac{\left( 6F\times C_{\mathrm{NH}_{3}}\times V \right)}{\left( 17\times Q \right)}\times100\%$$

Where C_NH3_ is the concentration of NH_3_ (mg/mL), V is the volume of electrolyte taken (mL), t is the duration of the chronoamperometry measurement (1 hours), A is the area of the electrocatalyst dropping on the carbon paper (cm^-2^), F is the Faraday constant (96487 C/mol), Q is the total charge (Coulomb).

The concentration of NH_4_^+^ in post-electrolysis catholyte was further quantified via ¹H NMR spectroscopy using maleic acid as an internal standard. Calibration curves were established with synthetic NH_4_^+^ standards (0-50 μM) prepared by dissolving (NH_4_)_2_SO_4_ in 0.1 M PBS. The sample preparation protocol is as follows: 2 mL of electrolyte sample was acidified with 2 mL 1 M H_2_SO_4_, 60 μL of the acidified solution was mixed with 20 μL maleic acid (3.6 mM), 20 μL 4 M H_2_SO_4_, and 500 μL DMSO-d_6_ in an NMR tube. The ¹H NMR spectra were acquired with 128 scans, 2 s relaxation delay, and water suppression (zgpr pulse sequence). The NH_4_^+^ concentration was determined by comparing the integrated peak area ratio of NH_4_^+^ (δ ≈ 6.9-7.1 ppm) to maleic acid (δ ≈ 6.3 ppm, singlet).

**Determination of hydroxylamine (NH_2_OH)**: To quantify NH_2_OH, the following procedure was employed: 1.0 mL PBS and 1% 8-hydroxylquinoline was combined with 1.0 mL catholyte (or standard NH_2_OH solution with known concentration). Under vigorous shaking, 1.0 mL of 0.1 M Na_2_CO_3_ was introduced to the mixture. The resulting solution was heated at 100°C for 1 minute. In the presence of NH_2_OH, the solution underwent a visible color change from light yellow to blue-green, with a characteristic absorption peak emerging at approximately 705 nm. By plotting the peak absorbance values against corresponding NH_2_OH concentrations, a calibration curve was established. This curve served as the basis for quantifying NH_2_OH concentrations in experimental samples. The FE for NH_2_OH was determined using the following equations:

$$\mathrm{FE}\left( \% \right)=\frac{\left( 4F\times C_{NH2OH}\times V \right)}{Q}\times100\%$$

**Determination of hydrazine (N_2_H_4_):** The Watt and Chrisp method were used to measure the N_2_H_4_ concentration in the electrolyte. The color reagent was made by mixing 300 mL of ethanol, 5.99 g of para-(dimethylamino) benzaldehyde, and 30 mL of concentrated HCl. Then, 4 mL of electrolyte was mixed with 1 mL of 1 M KOH, and 5 mL of color reagent. After sitting for 20 minutes at room temperature, the absorbance at 455 nm was measured using a UV-Vis spectrophotometer. A series of standard N_2_H_4_ solutions were used to create a calibration curve for quantifying N_2_H_4_.

**2.5 ^15^N Isotope Labelling Experiment.**

The ^1^H NMR spectroscopy was utilized for a ^15^N isotope labelling experiment to identify the nitrogen source for NO_2_RR. The reaction was conducted at a potential of -0.38 V vs. RHE for 1 hour. Two electrolyte solutions were prepared: one containing 0.1 M PBS and 0.05 M K^15^NO_2_, and the other with 0.1 M PBS and 0.05 M K^14^NO_2_. Then, 2 mL of the catholyte was collected and acidified with 1 mL of 1 M H_2_SO_4_. Subsequent steps included adding 60 μL of neutralized electrolyte, 20 μL of 3.6 mM maleic acid (internal standard), and 500 μL of d_6_-DMSO into an NMR tube. The sealed mixture underwent ^1^H NMR measurement to analyze the isotopic composition and identify the nitrogen source utilized in the NO_2_RR process.

**2.6 Differential Electrochemical Mass Spectrometry (DEMS) Measurements.**

The DEMS measurements were performed using a QAS 100 spectrometer (Ling Lu Instruments, Shanghai). The electrolyte, composed of 0.05 M KNO_2_ and 0.1 M PBS, was contained in a custom-made electrochemical cell. The working electrode was an Au foil coated with electrocatalysts, while the counter and reference electrodes were a Pt wire and an SCE, respectively. The I-t technique was applied at -0.38 V vs. RHE, and the corresponding mass signals were recorded. After each electrochemical test, when the mass signal returned to the baseline, the next cycle was initiated under the same conditions to minimize potential errors. This procedure was repeated for three consecutive cycles.

**2.7 Attenuated Total Reflection** - **Infrared Reflection Absorption Spectroscopy (ATR-IRAS) Measurement.**

The ATR-IRAS experiments were conducted using a Bruker INVENIO R spectrometer equipped with a liquid nitrogen-cooled mercury cadmium telluride A (MCT-A) detector. The custom-made spectro-electrochemical cell was provided by Ling Lu Instruments (Shanghai), and the measurements were controlled by a CHI 760E electrochemical workstation. For each experiment, 20 mL of ink containing the catalyst was dropped onto a polished Si wafer serving as the working electrode. The reference and counter electrodes were an SCE and a platinum wire, respectively. Data collection occurred at a resolution of approximately 30 seconds per spectrum. Measurements were taken simultaneously at -0.68 V vs. RHE for 1 hour or from the open circuit potential. Any interference from water or oxygen peaks was carefully removed from the data.

**2.8 Electron Paramagnetic Resonance (EPR) Measurement.**

The EPR measurements were carried out using an EPR200-PLUS model instrument (China Guoyi Quantum). The experimental conditions were set as follows: test temperature at 298 K, modulation frequency at 100 kHz, microwave power at 1 mW, microwave frequency at 9.52 GHz, scanning center at 3398 G, scanning width at 100 G, and modulation amplitude at 1 G.

**2.9 Energy Efficiency Calculation**

The EE (Energy Efficiency) and the SEC (specific energy consumption (kWh/kg NH_3_)) are calculated using the following formula.

EE (%) = (Theoretical energy required for NH₃ synthesis) / (Actual energy input) × 100%.

EE (%) = $\frac{0.280245kJ/\mathrm{cm}^{2}}{0.377kJ/\mathrm{cm}^{2}}$ ×100%

The theoretical energy for NH_3_ synthesis is derived from the Gibbs free energy change of nitrite reduction to NH_3_ (ΔG = -357 kJ/mol NH_3_), while the actual energy input is calculated as "cell voltage (V) × current (A) × time (h)".

Total current = Current density × Electrode area

NH_3_ Partial Current = I_total_ × FE

Annual Power Consumption of the Electrolyzer = *I*_total_×*V*_cell_

Annual NH_3_ Production = 552.16×10^-6^ kg h^-1^ cm^-2^ ×4 cm^2^×8760 h= 19.41 kg/year

Specific Energy Consumption (SEC) = Annual production/Annual energy=13.9 kWh/kg NH_3_

**3.0 Computational Details.**

The first-principles calculations were performed based on density functional theory and implemented in the Vienna Ab initio Simulation package (VASP).^[1]^ The projector-augmented wave (PAW) pseudopotential was used and the generalized gradient approximation of the Perdew-Burke-Ernzerhof (PBE-GGA) method was employed in the calculations.^[2]^ A 400 eV cut off energy was set for the plane-wave basis set. The convergence accuracy for the energy and force was set to 10^−4^ eV and 0.01 eV Å^−1^. A 2×2×1 Monkhorst-Pack k-mesh in the Brillouin zone was used for the optimization.^[3]^ The van der Waals interactions were described by an empirical correction in Grimme's scheme (DFT-D3).^[4]^ To obtain the ideal electronic structure, the U values of the Zn 3d orbitals were set to 11.6 eV, respectively. At the same time, U = 3 eV was used to consider the Coulomb interaction effect on Cu atoms. There was a vacuum of 15 Å in both directions perpendicular to the periodic direction to avoid the interactions between adjacent cells. The Gibbs free energy (ΔG) was calculated to evaluate performance. The free energy ΔG is defined as ΔG = ΔE + ΔE_ZPE_ − TΔS, where ΔE, ΔE_ZPE_, and ΔS are the adsorption energy, the zero-point energy (ZPE), and the entropy differences between the adsorbed state and the gas phase, respectively. During structural optimizations, the gamma point in the Brillouin zone was used for k-point sampling. Spin-polarized calculations were performed for this calculation. The adsorption energy E_ads_ is calculated as: E_ads_ = E_total_ - E_slab_- E_adsorbate_. E_total_ is the total energy of an optimized slab with the adsorbed, E_slab_ is the energy of a relaxed and clean slab, and E_adsorbate_ is the energy of an adsorbate molecule.

# 3. Figures and Tables

**
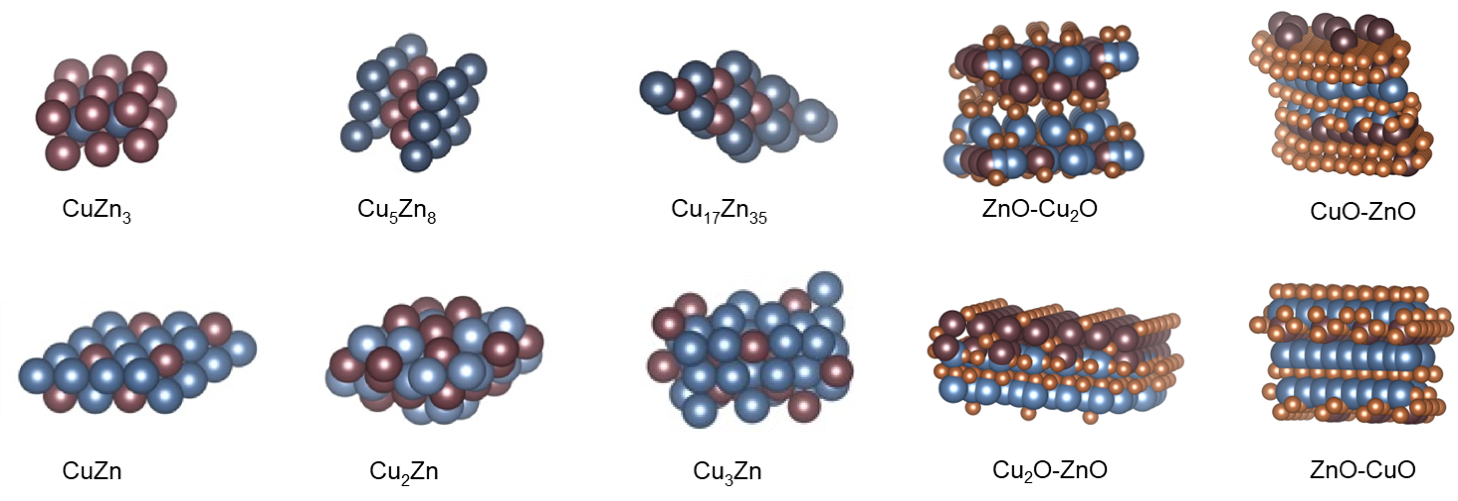
Figure S1****.** Six Cu-Zn alloys and four heterostructures with different crystal structures (blue and red represent Zn and Cu atoms, respectively).

**
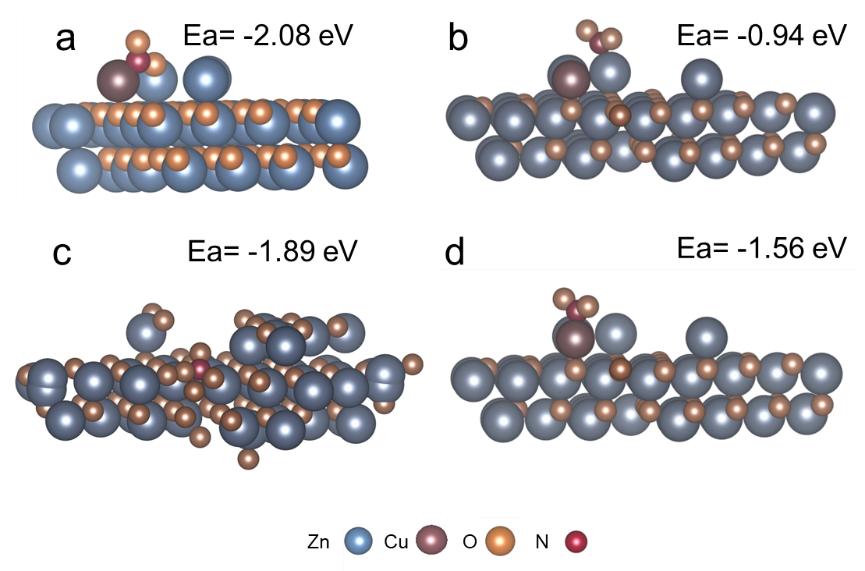
**

**Figure S2**. The structures and adsorption energy of *NO_2_ across varying sites of Cu-ZnO, (a) Cu and Zn bridge sites with oxygen vacancies, (b) Zn site, (c) oxygen vacancy site, (d) Cu site.

**
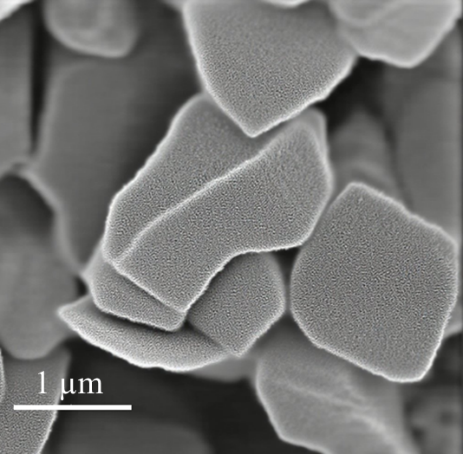
**

**Figure S3.** SEM image of Cu-ZnO.


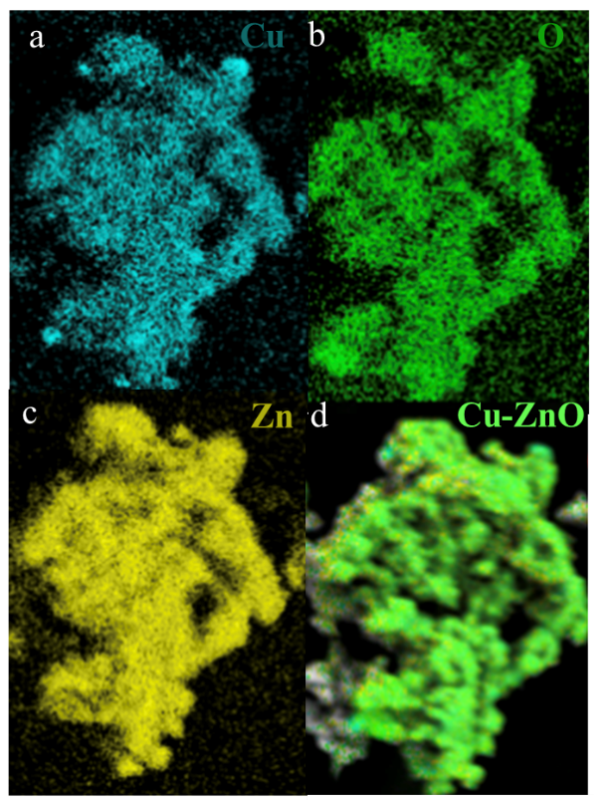


**Figure S4.** EDS mapping of Cu-ZnO catalyst, showing the elemental dispersion of Cu (blue), O (green) and Zn (yellow).


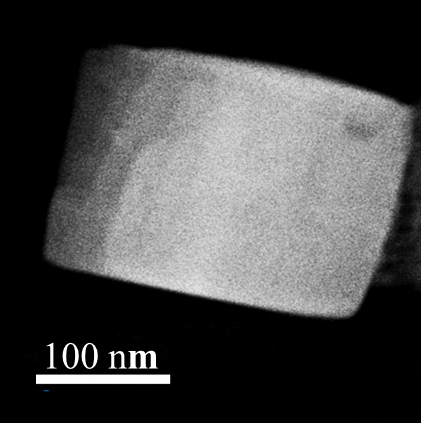


**Figure S5.** TEM image of Cu-ZnO.


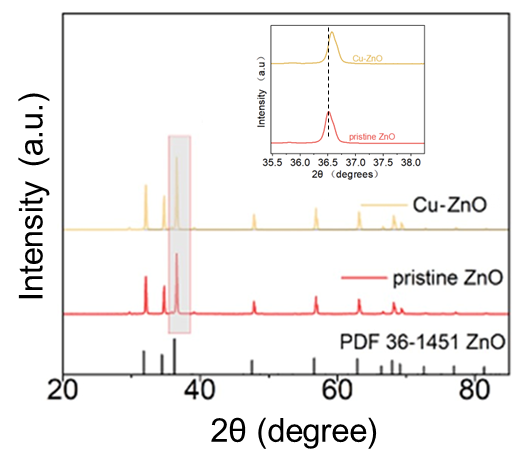


**Figure S6**. XRD pattern of Cu-ZnO and pristine ZnO, inset is the magnified view.

A shift in the diffraction peaks towards higher angles (2θ) is observed in the XRD pattern of Cu-ZnO. This phenomenon indicates that copper is incorporated into the interstitial spaces within the lattice, which is due to the considerably smaller ionic radius of Cu^2+^ (0.73 Å) compared to that of Zn^2+^ (0.74 Å). The Cu-ZnO sample exhibits a shift in the diffraction peaks towards higher angles (2θ), which suggests a contraction of the crystal lattice.


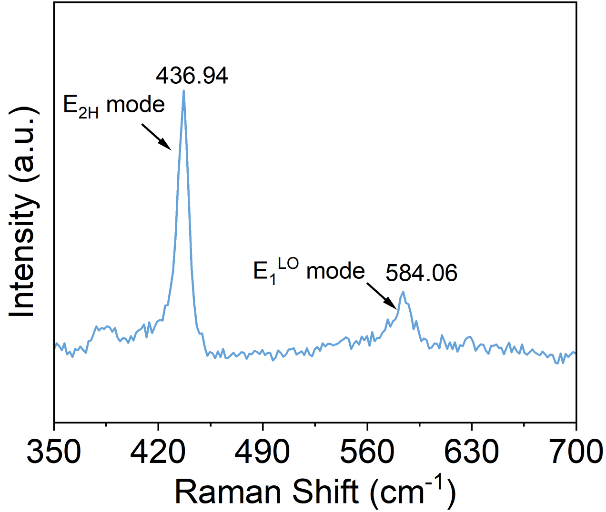


**Figure S7.** Raman spectra showing the structure of Cu-ZnO.

Cu doping into ZnO shifts the non-polar E_2H_ mode from 431 to 437 cm^−1^. This peak shift is likely assigned to lattice distortion induced by Cu doping within the ZnO crystal. Furthermore, the Raman spectra of Cu-ZnO in 200 to 800 cm^−1^ range remain similar to those of pure ZnO , with the characteristic ZnO peak near 436.94 cm^−1^.


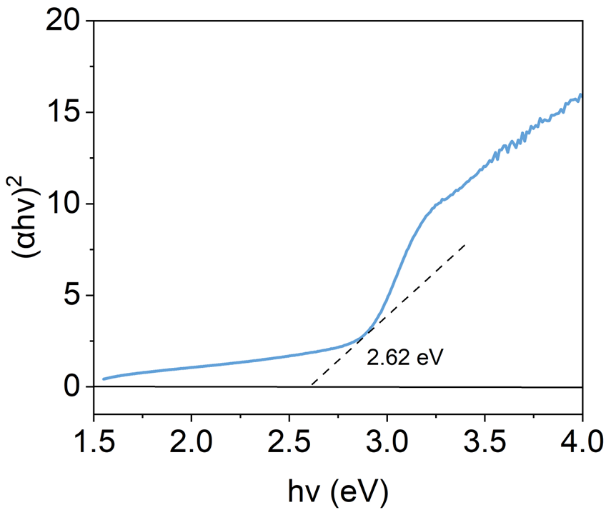


**Figure S8.** The Tauc plot illustrates the optical band gap (E_g_)of Cu-ZnO.

In the Tauc plot, the bandgap of Cu doped ZnO was found to be 2.62 eV, compared to the 3.37 eV for pristine ZnO. This significant reduction (0.75 eV) can be ascribed to the incorporation of. The hybridization of the closely matching energy levels of Cu's 3d orbitals with the O's 2p orbitals could be a contributing factor to the observed band gap reduction. As a result, the formation of the CuO phase can cause a pronounced redshift of approximately 0.75 eV in the optical properties.


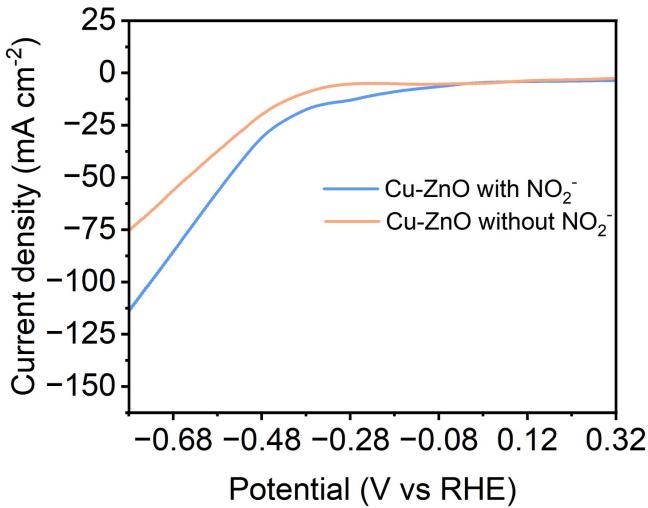


**Figure S9.** The LSV curve of Cu-ZnO catalyst.


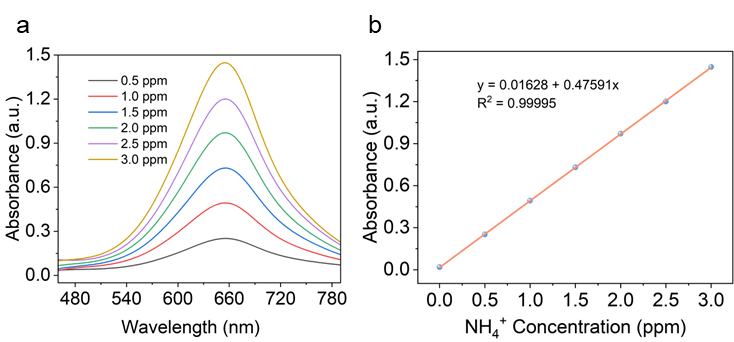


**Figure S10.** (a) UV-Vis absorption spectra of NH_4_Cl standard solutions. (b) Corresponding calibration curve used for calculation of NH_3_ concentration.


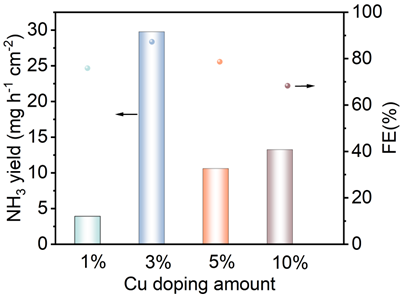


**Figure S11.** FE and NH_3_ yield of Cu-ZnO with varying Cu doping amounts, bar charts represent the NH_3_ yield, while scatter plots represent the FE.


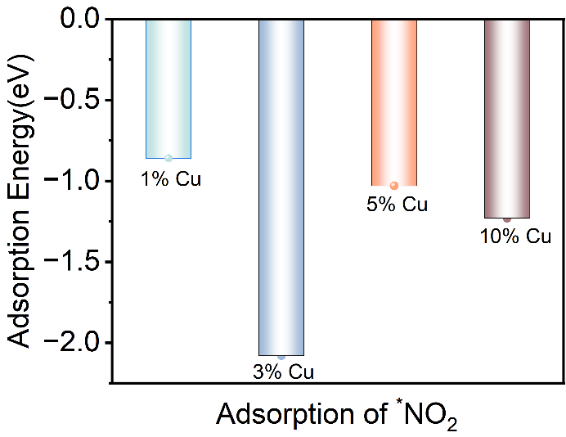


**Figure S12**. The adsorption energy of *NO_2_ over Cu-ZnO with varying Cu doping amounts.


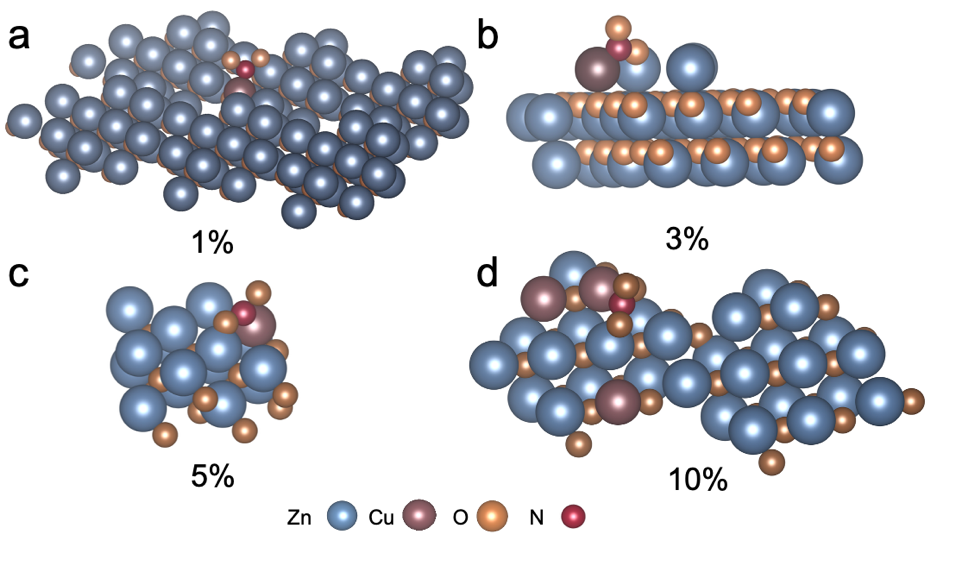


**Figure S13**. The structures of Cu-ZnO with absorbed *NO_2_ with varying Cu doping amounts. (a)1%, (b) 3%, (c) 5% and (d) 10%.


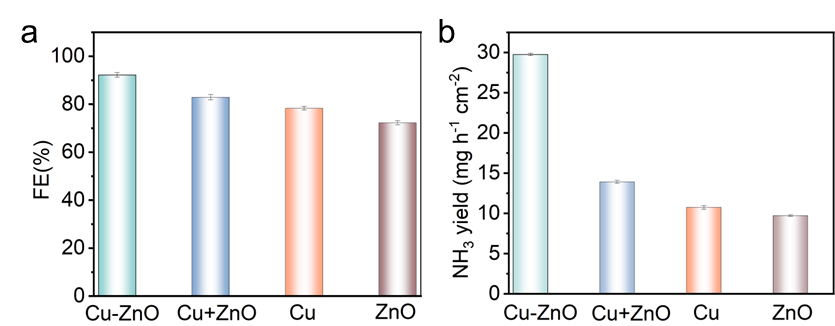


**Figure S14**. (a) FE and (b) NH_3_ yield of Cu-ZnO, Cu, physically mixed Cu+ZnO, and ZnO.


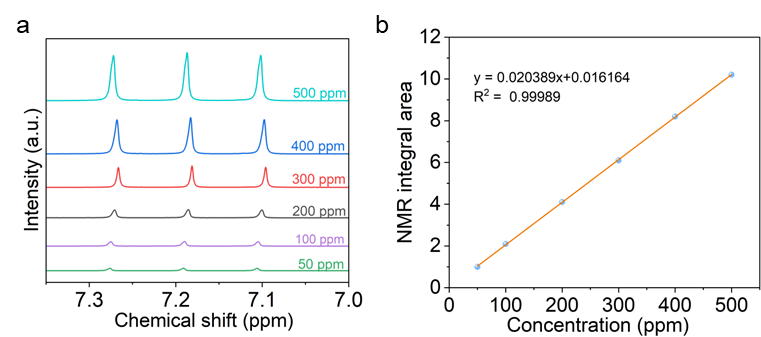


**Figure S15.** (a) ^1^H-NMR spectra of different concentrations NH_4_Cl for calibration. (b) Corresponding calibration curve of NMR integral area (NH_4_^+^-N/C_4_H_4_O_4_) against N (NH_4_^+^) concentration.

The concentration of NH_4_^+^ can be quantitatively determined by ^1^H-NMR. The NMR with internal standards (maleic acid, C_4_H_4_O_4_). The triple peaks were assigned to NH_4_^+^. The integral area is proportional to the amount of H.


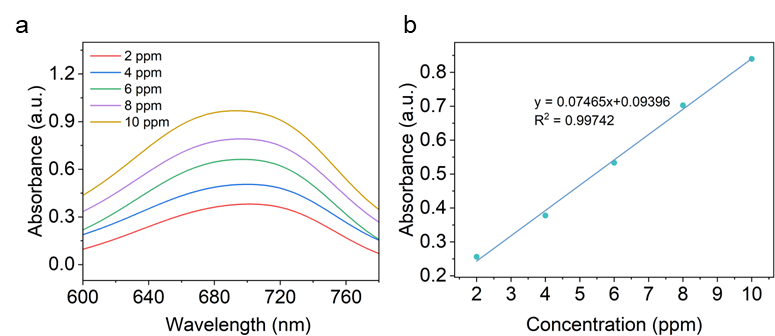


**Figure S16.** (a) UV-Vis absorption spectra of NH_2_OH standard solutions. (b) Calibration and corresponding calibration curve used for calculation of NH_2_OH concentrations.


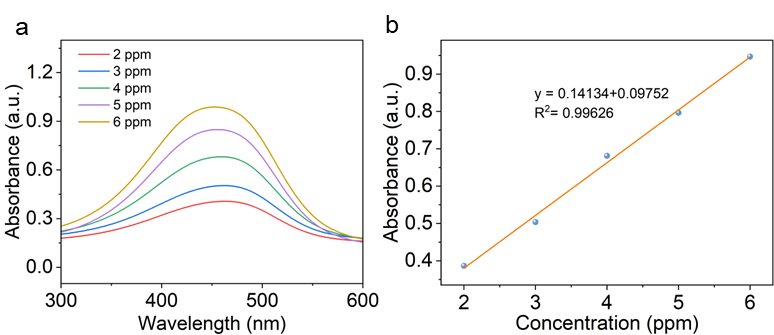


**Figure S17.** (a) UV-Vis absorption spectra of N_2_H_4_ standard solutions. (b) Calibration and corresponding calibration curve used for calculation of N_2_H_4_ concentrations.


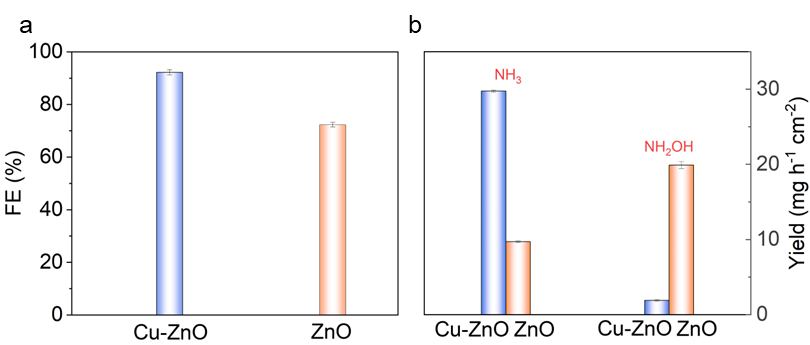


**Figure S18**. (a) The NH_3_ FE of Cu-ZnO and ZnO. (b) NH_3_ and NH_2_OH yield of Cu-ZnO and ZnO.


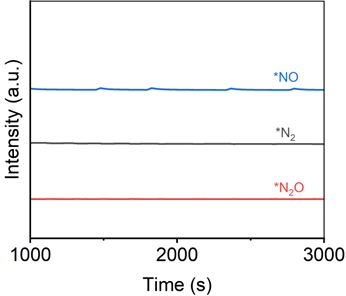


**Figure S19**. Online differential electrochemical mass spectrometry (DEMS) spectra over Cu-ZnO.


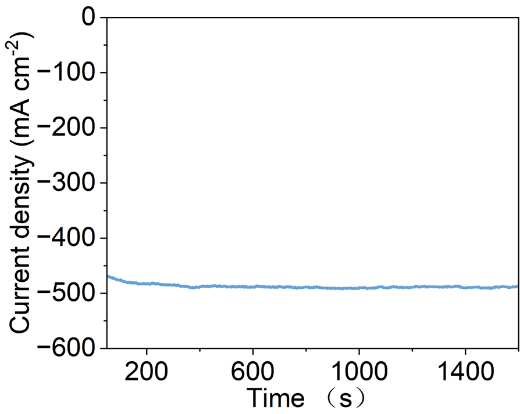


**Figure S20.** The i-t curve for NO_2_RR in the MEA reactor.


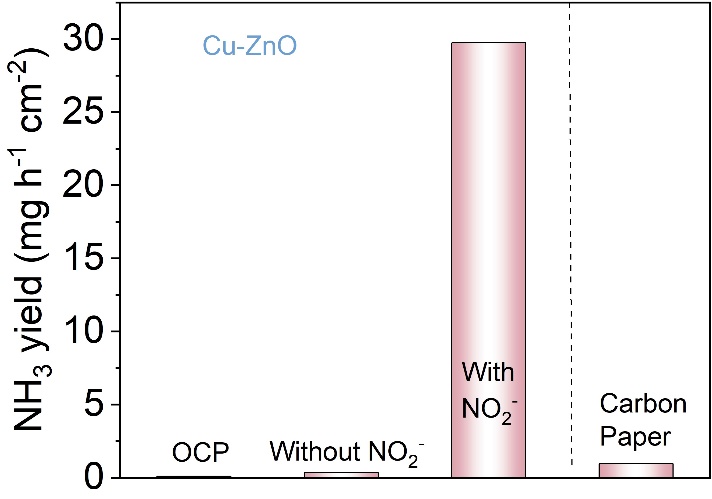


**Figure S21.** The NH_3_ yield for NO_2_RR in the H-cell, OCP stands for open circuit voltage, and CP stands for carbon paper without catalysts.

The experimental conditions are as follows: (1) open-circuit potential (OCP) with 0.5 M KNO_2_ and 0.1 M PBS electrolyte using the Cu-ZnO catalyst; (2) without NO_2_^-^, using 0.1 M PBS electrolyte and the Cu-ZnO catalyst at -0.38 V vs RHE; (3) with NO_2_^-^, using 0.5 M KNO_2_ and 0.1 M PBS electrolyte and the Cu-ZnO catalyst at -0.38 V vs RHE; (4) using bare carbon paper with 0.5 M KNO_2_ and 0.1 M PBS electrolyte at -0.38 V vs RHE.


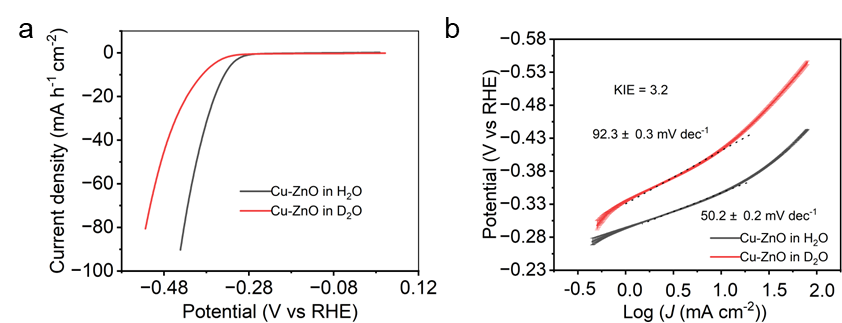


**Figure S22.** (a) The polarization curves of Cu-ZnO in H_2_O and D_2_O, (b) Tafel plots derived from the polarization curves in a.


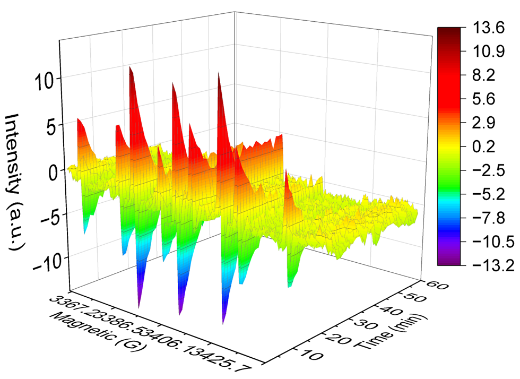


**Figure S23.** The in-situ EPR signals for NO_2_RR.

The signals of active *H decrease over time, indicating their utilization in the proton coupling process.


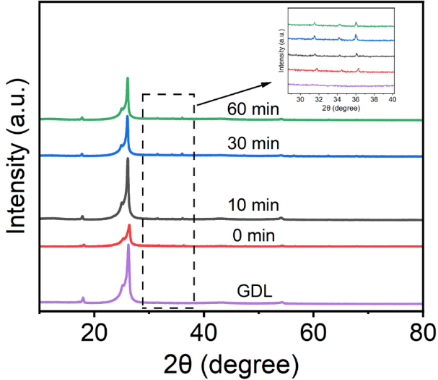


**Figure S24**. The XRD patterns of Cu-ZnO catalyst coated on the gas diffusion layer (GDL) during the electrochemical test.


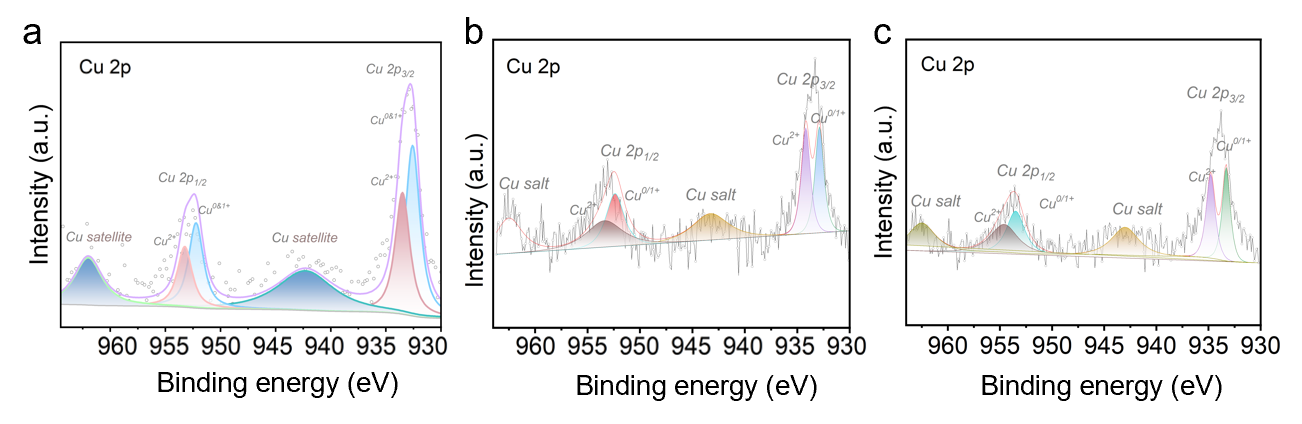


**Figure S25**. The Cu 2p XPS pattern of Cu-ZnO during the electrochemical test. (a) 0 min, (b) 30 min, (c) 60 min.


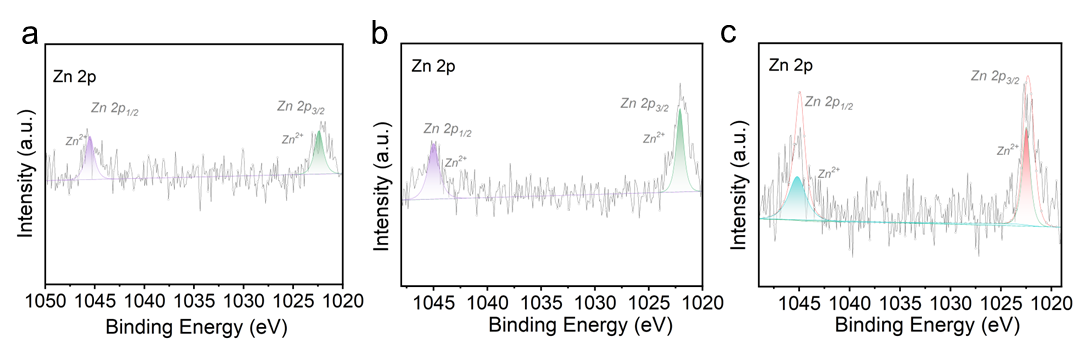


**Figure S26.** The Zn 2p XPS pattern of Cu-ZnO during the electrochemical test. (a) 0 min, (b) 30 min, (c) 60 min.


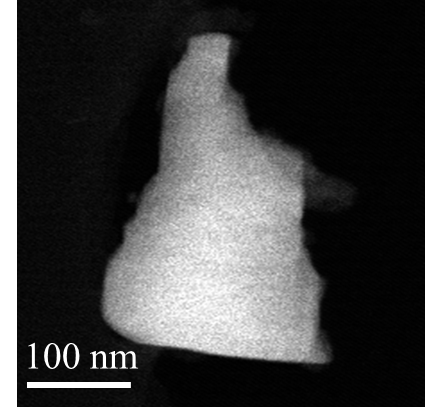


**Figure S27**. The TEM image of Cu-ZnO after the electrochemical test.


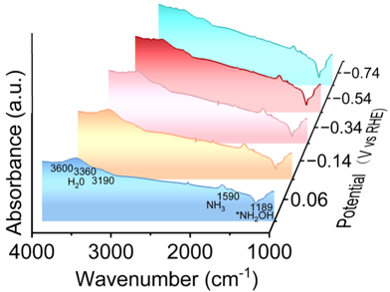


**Figure S****28.** The in-situ ATR-IRAS measurement under different applied potentials.


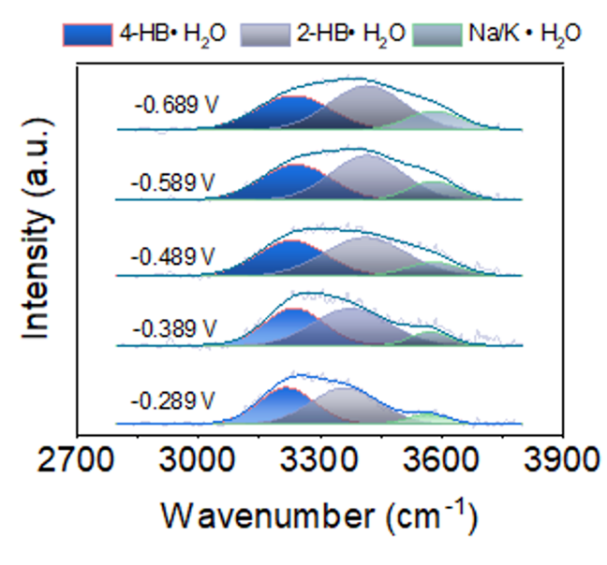


**Figure S29.** The corresponding Gaussian-fitted peaks revealing the three O-H stretching modes of interfacial water on Cu-ZnO for NO_2_RR.


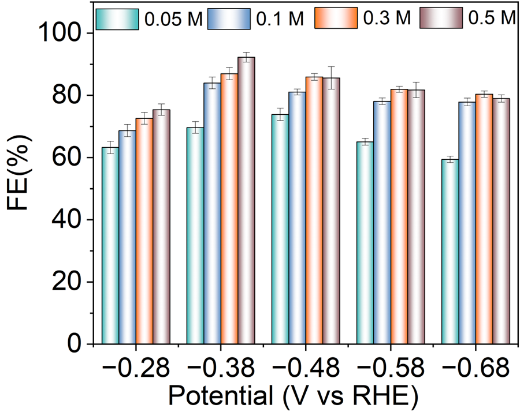


**Figure S30.** FE of Cu-ZnO in a simulated wastewater system containing representative concentrations of nitrite (0.05-0.5 M KNO_2_) and common inorganic ions (e.g., HPO_4_^2^⁻/H_2_PO_4_⁻).


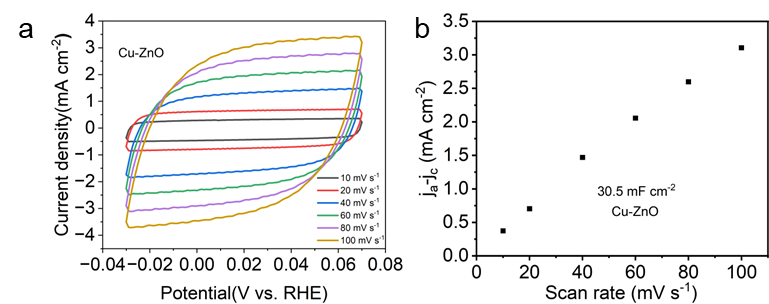


**Figure S31.** (a) The CV curves of Cu-ZnO at various scan rates, (b) corresponding calibration curve used for the calculation of C_dl_.


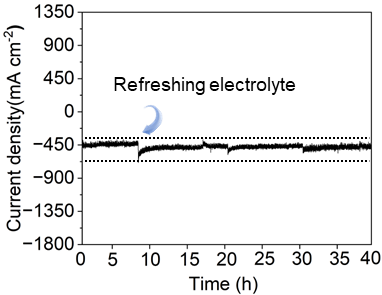


**Figure S32.** The stability test of Cu-ZnO in the MEA configuration at -1.6 V (electrolyzer cell voltage).


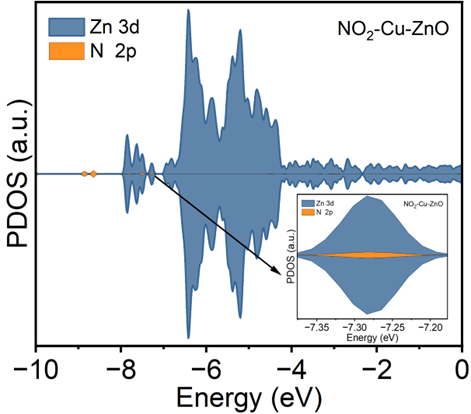


**Figure S33.** The projected density of states (PDOS) after NO_2_^−^ adsorption.


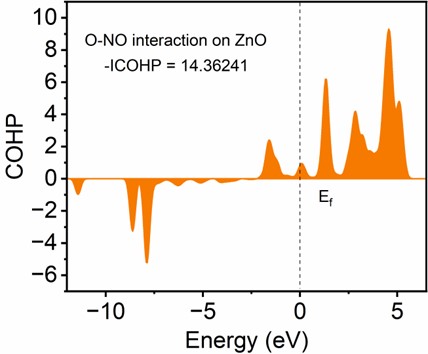


**Figure S34.** The COHP of the O-NO interaction on ZnO.


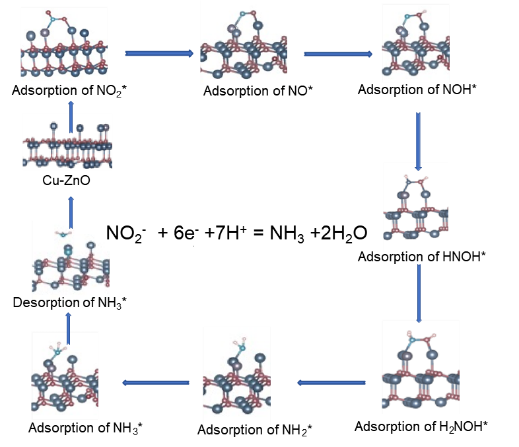


**Figure S35.** The reaction pathway of NO_2_RR.

The reaction pathway of NO_2_RR was described as follows:

* + NO_2_^-^ + H_2_O + e^-^ → *NO + 2OH^-^ (1)

*NO + H_2_O + e^-^ → *NOH + OH^-^ (2)

*NOH + H_2_O + e^-^ → *HNOH + OH^-^ (3)

*HNOH + H_2_O+ e^-^ → *NH_2_OH + OH^-^ (4)

*NH_2_OH + e^-^ → *NH_2_ + OH^-^ (5)

*NH_2_ + H_2_O + e^-^ → *NH_3_ + OH^-^ (6)

*NH_3_^-^ → NH_3_ + * (7)

where * represents the active sites on surface of slab models. *i represents the adsorption

configuration of intermediate i on slab models.


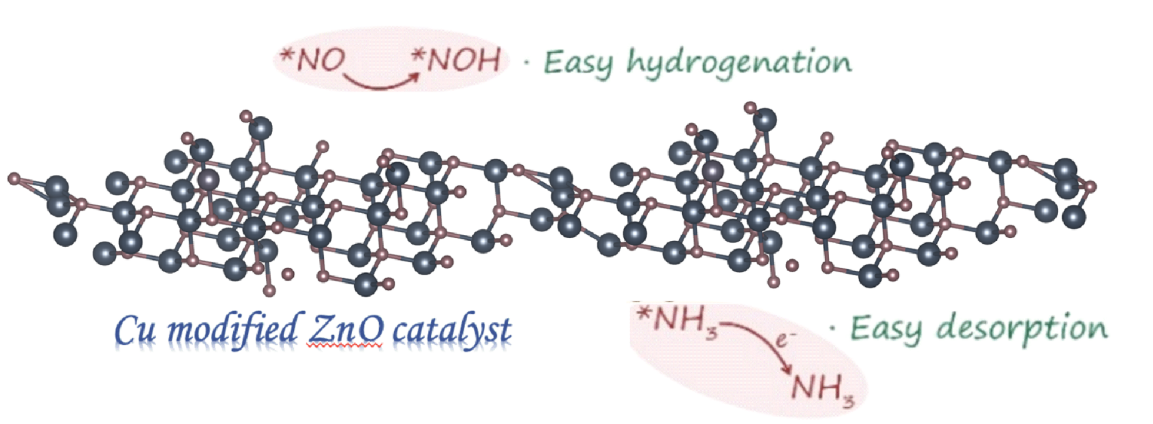


**Figure S36.** The illustration showing the easy hydrogenation and desorption of Cu-ZnO.

**Table S1.** The activation and reaction energy of H_2_O to *H on various metal surfaces.

| **Metal Surfaces** | **Activation** **Energy (eV)** | **Reaction** **Energy (eV)** |
| --- | --- | --- |
| Au111 | 2.00 | 1.56 |
| Ag111 | 1.82 | 0.95 |
| Ag211 | 1.39 | 0.77 |
| Pt111 | 0.90 | 0.69 |
| Cu111 | 1.33 | 0.36 |
| Rh111 | 0.95 | 0.23 |
| Pd111 | 1.01 | 0.22 |
| Pd211 | 1.00 | 0.07 |
| Ru111 | 0.95 | -0.04 |
| Cu211 | 0.80 | -0.10 |
| Ni111 | 0.91 | -0.19 |
| Rh211 | 0.49 | -0.86 |
| Re111 | 0.52 | -0.88 |
| Ni211 | 0.35 | -0.92 |
| Ir211 | 0.24 | -1.06 |
| Fe211 | 0.36 | -1.20 |
| Ru211 | 0.01 | -1.22 |
| Mn211 | 0.26 | -1.43 |
| Mn111 | 0.55 | -1.51 |

**Table S2**. EXAFS fitting parameters at the Cu K-edge for Cu-ZnO, CuO, Cu_2_O and Cu foil.

| **sample name** | **bond** | **length** | **CN** | **ΔE** | **σ^2^ (10^-3^)** | **R-factor** |
| --- | --- | --- | --- | --- | --- | --- |
| Cu-ZnO | Cu-O | 1.98 (0.03) | 33.8 (0.3) | 8.4 (0.8) | 8.1 (0.7) | 0.40% |
| Cu foil | Cu-Cu | 2.57 (0.03) | 412* | 5.4(0.6) | 4.8 (0.3) | 0.90% |
| CuO | Cu-O | 1.96 (0.02) | 4* | 1.5 (0.5) | 3.7 (0.6) | 0.70% |
| Cu_2_O | Cu-O | 1.85 (0.02) | 4* | 6.1 (0.4) | 3.5 (0.5) | 0.40% |

CN is the coordination number; R is the distance between absorber and backscatter atoms; σ^2^ is the Debye-Waller factor to account for both thermal and structural disorders; ΔE is the inner potential correction (edge-energy shift); R factor indicates the goodness of the fit.

**Table S3.** The comparison of NH_3_ yield and FE with reported literatures.

| **NO_2_RR catalyst** | **Supporting electrolyte** | **NH_3_ yield**  **(mg h^−1^ cm^−2^)** | **Electrolyzer** | **FE, %** | **Potential**  **(V vs RHE)** | **Ref** |
| --- | --- | --- | --- | --- | --- | --- |
| **Cu-ZnO** | **0.1 M PBS** | **2.975** | **H-cell** | **92.2%** | **-0.38 V** | **This Work** |
| **Cu-ZnO** | **0.1 M PBS** | **133.5** | **Flow-cell** | **89.1%** | **-0.38 V** | **This Work** |
| **Cu-ZnO** | **0.1 M PBS** | **552.2** | **MEA** | **87.9%** | **-0.38 V** | **This Work** |
| Cu_3_P NA/CF | 0.1 M PBS | 1.626 | H-cell | 91.2±2.5% | -0.5 V | [5] |
| Cu@Cu_2_O | 0.5 M Na_2_SO_4_ | 0.5109 | Flow-cell | 27% | -1.0 V | [6] |
| Ag@NiO/CC | 0.1 M NaOH | 5.751 | H-cell | 97.7 % | -0.7 V | [7] |
| ITO@TiO_2_/TP | 0.5 M LiClO_4_ | 6.992 | H-cell | 82.6% | -0.5 V | [8] |
| V–TiO_2_/TP | 0.1 M NaOH | 9.194 | Flow-cell | 93.2% | -0.7 V | [9] |
| Cu_3_P@TiO_2_/TP | 0.1 M NaOH | 17.60 | H-cell | 97.1% | -0.8 V | [10] |
| P-TiO_2_/TP | 0.1 M Na_2_SO_4_ | 9.534 | H-cell | 90.6% | -0.6 V | [11] |
| Cu foam@Cu_2_O | 0.1 M PBS | 7.511 | H-cell | 94.2% | -0.6 V | [12] |
| CoP nanoarray | 0.1 M PBS | 0.1330 | H-cell | 90.2% | -0.2 V | [13] |
| Fe-Co_3_O_4_ NA/TM | 0.1 M PBS | 0.6240 | H-cell | 95.5% | -0.7 V | [14] |
| Co_3_O_4_/Co-h | 0.1 M Na_2_SO_4_ | 4.430 | Flow-cell | 88.7 % | -0.8 V | [15] |
| Co_3_O_4_/NiO | 0.5 M Na_2_SO_4_ | 6.510 | MEA | ~99.4% | -0.6 V | [16] |
| CoP-CNS | 1M NaOH | 143.9 | MEA | 88.6% | -1.03 V | [17] |
| MnCuO_x_-H | 1 M KOH | 9.400 | MEA | 86.4 % | -0.63 V | [18] |

**References**

[1] J. Hafner, J. Comput. Chem. **2008**, *29*, 2044-2078.

[2] P. E. Blöchl, **Phys. Rev. B. 1994,** *50*, 17953-17979.

[3] S. Grimme, J. Comput. Chem. **2006**, *27*, 1787-1799.

[4] H. J. Monkhorst, J. D. Pack, **Phys. Rev. B.** **1976,** 13, 5188.

[5] J. Liang, B. Deng, Q. Liu, G. Wen, Q. Liu, T. Li, Y. Luo, A. A. Alshehri, K. A. Alzahrani, D. Ma, X. Sun, Green Chem. **2021**, 23, 5487-5493.

[6] S. Yeon, S. J. Lee, J. Kim, T. Begildayeva, A. Min, J. Theerthagiri, M. L. A. Kumari, L. M. C. Pinto, H. Kong, M. Y. Choi, Environ. Res. **2022**, 215, 114154.

[7] Q. Liu, G. Wen, D. Zhao, L. Xie, S. Sun, L. Zhang, Y. Luo, A. Ali Alshehri, M. S. Hamdy, Q. Kong, X. Sun, J. Colloid Interface Sci. **2022**, 623, 513-519.

[8] S. Li, J. Liang, P. Wei, Q. Liu, L. Xie, Y. Luo, X. Sun, eScience **2022**, 2, 382-388.

[9] H. Wang, F. Zhang, M. Jin, D. Zhao, X. Fan, Z. Li, Y. Luo, D. Zheng, T. Li, Y. Wang, B. Ying, S. Sun, Q. Liu, X. Liu, X. Sun, Mater. Today Phys. **2023**, 30, 100944.

[10] Z. Cai, D. Zhao, X. Fan, L. Zhang, J. Liang, Z. Li, J. Li, Y. Luo, D. Zheng, Y. Wang, T. Li, H. Yan, B. Ying, S. Sun, A. A. Alshehri, H. Yan, J. Xu, Q. Kong, X. Sun, Small **2023**, 19, 2300620.

[11] L. Ouyang, X. He, S. Sun, Y. Luo, D. Zheng, J. Chen, Y. Li, Y. Lin, Q. Liu, A. M. Asiri, X. Sun, J. Mater. Chem. A **2022**, 10, 23494-23498.

[12] Q. Chen, X. An, Q. Liu, X. Wu, L. Xie, J. Zhang, W. Yao, M. S. Hamdy, Q. Kong, X. Sun, Chem. Commun. **2022**, 58, 517-520.

[13] G. Wen, J. Liang, Q. Liu, T. Li, X. An, F. Zhang, A. A. Alshehri, K. A. Alzahrani, Y. Luo, Q. Kong, X. Sun, Nano Res. **2022**, 15, 972-977.

[14] P. Wei, J. Liang, Q. Liu, L. Xie, X. Tong, Y. Ren, T. Li, Y. Luo, N. Li, B. Tang, A. M. Asiri, M. S. Hamdy, Q. Kong, Z. Wang, X. Sun, J. Colloid Interface Sci. **2022**, 615, 636-642.

[15] F. Zhao, G. Hai, X. Li, Z. Jiang, H. Wang, Chem. Eng. J. **2023**, 461, 141960.

[16] J. Xu, S. Zhang, H. Liu, S. Liu, Y. Yuan, Y. Meng, M. Wang, C. Shen, Q. Peng, J. Chen, X. Wang, L. Song, K. Li, W. Chen, Angew. Chem. Int. Ed. **2023**, 62, e202308044.

[17] K. Fan, W. Xie, J. Li, Y. Sun, P. Xu, Y. Tang, Z. Li, M. Shao, Nat. Commun. **2022**, 13, 7958.

[18] D. Jang, J. Maeng, J. Kim, H. Han, G. H. Park, J. Ha, D. Shin, Y. J. Hwang, W. B. Kim, Appl. Surf. Sci. **2023**, 610, 155521.
